# Compact and ultracompact spectral imagers: technology and applications in biomedical imaging

**DOI:** 10.1117/1.JBO.28.4.040901

**Published:** 2023-04-05

**Authors:** Minh H. Tran, Baowei Fei

**Affiliations:** a University of Texas at Dallas, Department of Bioengineering, Richardson, Texas, United States; b University of Texas Southwestern Medical Center, Department of Radiology, Dallas, Texas, United States; c University of Texas at Dallas, Center for Imaging and Surgical Innovation, Richardson, Texas, United States

**Keywords:** camera, compact, hyperspectral imaging, multispectral imaging, spectral imaging

## Abstract

**Significance:**

Spectral imaging, which includes hyperspectral and multispectral imaging, can provide images in numerous wavelength bands within and beyond the visible light spectrum. Emerging technologies that enable compact, portable spectral imaging cameras can facilitate new applications in biomedical imaging.

**Aim:**

With this review paper, researchers will (1) understand the technological trends of upcoming spectral cameras, (2) understand new specific applications that portable spectral imaging unlocked, and (3) evaluate proper spectral imaging systems for their specific applications.

**Approach:**

We performed a comprehensive literature review in three databases (Scopus, PubMed, and Web of Science). We included only fully realized systems with definable dimensions. To best accommodate many different definitions of “compact,” we included a table of dimensions and weights for systems that met our definition.

**Results:**

There is a wide variety of contributions from industry, academic, and hobbyist spaces. A variety of new engineering approaches, such as Fabry–Perot interferometers, spectrally resolved detector array (mosaic array), microelectro-mechanical systems, 3D printing, light-emitting diodes, and smartphones, were used in the construction of compact spectral imaging cameras. In bioimaging applications, these compact devices were used for *in vivo* and *ex vivo* diagnosis and surgical settings.

**Conclusions:**

Compact and ultracompact spectral imagers are the future of spectral imaging systems. Researchers in the bioimaging fields are building systems that are low-cost, fast in acquisition time, and mobile enough to be handheld.

## 1 Introduction and Motivation

Light interacts with objects through various means of scattering, absorption, reflection, and transmission. In the late 18th to early 19th century, studies involving light emitted from chemical flames and celestial bodies demonstrated that each chemical compound has a different fingerprint in its interactions with electromagnetic radiation. Nowadays, using instruments called spectrometers or spectrographs, the incoming radiation can be separated by wavelengths, and the resulting spectrum can be matched to determine the types and amount of chemicals. If spectrometers typically analyze the compound within a limited field of view, spectral imaging expands the field of view to include the spatial morphology of the subject. Spectral imaging is more powerful than spectrometry in understanding not just the types and amount but also the spatial distribution of chemicals.^1^ As such, spectral imaging is used in almost every application that requires imaging, such as satellite,^2^ agriculture,^3^ food science,^4^ and art.^5^ In biomedical imaging, spectral imaging has some advantages over regular color images. The amount of spectral and morphology information provides better understanding about physiological processes, which RGB and spectroscopy alone cannot achieve.^6^ Both Li et al.^6^ and Lu and Fei^7^ produced seminal literature reviews that discuss the different processes spectral imaging can reveal and help measure, such as metabolic processes; retinal oxygen saturation; tumors on the surface of skin, tongue, and mucosa; and ischemia in the intestine and the brain. Because spectral imaging mostly captures the reflected and scattered light in the nonionizing wavelengths, invasiveness and potential harm are minimal.

The motivation for compact and lightweight spectral camera systems came from remote sensing. Smaller and lighter cameras meant more space for other instruments. As spectral imaging was adapted to other fields, compact spectral cameras proved useful because they enabled on-the-spot sample acquisitions and analysis without cumbersome setup. Since the first generations of spectral cameras in the 1970s, innovations in compact spectrometry, manufacturing processes, material sciences, and computations have enabled compact and ultracompact spectral imaging systems.^8^ Innovations came from both industry and academia: while compact commercial devices were developed using proprietary solutions, many devices used within academia relied on low-cost, commercial-off-the-shelf (COTS) components. Since the last review of medical hyperspectral imaging from our research group,^7^ several new applications of spectral imaging in the medical and biological field became possible due to developments of compact and ultracompact spectral cameras. And yet, spectral imaging devices are still not widely used in the biomedical field.^6^^,^^9^ With this review paper, we hope that researchers can (1) understand the technological trends of upcoming spectral cameras, (2) understand new specific applications that portable spectral imaging unlocked, and (3) evaluate proper spectral imaging systems for their specific applications. Section 4 reviews the acquisition methods. Section 5 discusses components that enable the miniaturization of spectral cameras. In Sec. 6, we focus on a special subset of compact spectral cameras, which are spectral cameras that are both compact and low-cost, built using off-the-shelf components and low-cost manufacturing processes. In Sec. 7, we provide specific applications of compact spectral cameras in biomedical research. Finally, in Sec. 8 we provide extended discussion on the future of compact spectral cameras, in terms of engineering and biomedical applications.

## 2 Scope and Methodology

In this paper, the term “spectral imager” refers to both hyperspectral imagers (HSIs) and multispectral imagers (MSIs). In the early years of spectral imaging research, the term “imaging spectrometer” was also common.^10^^–^^15^ In general, MSIs capture <20 bands of wavelengths, whereas HSIs can capture 20 to hundreds of bands.^6^ Some literature uses the term “ultraspectral imaging,” which refers to systems that collect hundreds to thousands of bands.^16^ Spectral imaging captures light reflected, scattered, and fluoresced from a sample, as in conventional imaging. We do not cover other imaging modalities that also rely on the spectral response of tissues, such as laser speckle contrast imaging (LSCI),^17^ Raman spectroscopy,^18^ and optical coherence tomography.^19^ Readers should not confuse spectral imaging with multispectral photoacoustic imaging, which uses the formation of sound waves following light absorption to image at different wavelengths.^20^ We only discuss the cameras using digital sensors, as research in analog spectral cameras is almost nonexistent. Finally, the definition of “compact” as used in the literature varies depending on the field. Table 1 displays dimensions and weights for systems that meet our definition of “compact”: without external lens or cables, these systems weigh no more than 5 kg (∼11  lbs). We also define “ultracompact” cameras as systems that weigh <500  g (∼1.1  lbs). For comparison, commercially high-end digital single-lens reflex (DSLR) cameras typically weigh <2.5  kg (∼5.5  lbs), midrange commercial webcams weigh 80 to 100 g (∼3 to 4 oz), and modern handheld spectrometers usually weigh no more than 1 kg (∼2  lbs).^8^ Our findings are summarized in Table 1. In this paper, we use the notation h×w×λ to show the pixel raster size of the hypercube. h and w refer to the height and width in the spatial dimension, and λ refers to the number of bands in the spectral dimension.

**Table 1:** Compilation of compact spectral imaging systems used in medical and biological applications. Cust., customized or commercial-off-the-shelf systems; Comm., commercialized systems that can be purchased; LED, light-emitting diode; PGP, prism-grating-prism; AOTF, acousto-optic tunable filter; IMS, image mapping spectrometer; LCTF, liquid crystal tunable filter; FPI, Fabry–Perot interferometer; SRDA, spectrally resolved detector array; CTIS, computed tomographic imaging spectrometer; SFDI, spatial frequency-domain imaging; CCD, charge-coupled device; CMOS, complementary metal oxide semiconductors; CVD, cardiovascular disease; AMD, age-related macular degeneration; E, *ex vivo*; I, *in vivo*; H, human; A, animal; P, phantom. Weight refers to the weight of the camera and the lens only, not including other optical systems that may be used in the acquisition of biological samples.

	System specifications							Biomedical application					
Year	System	Acquisition mode	Dispersive device	Range (nm)	# Bands	Sensor	Weight (kg)	Procedure	Disease/process	Organs	I/E	Subject	Ref.
2001	Cust.	Spectral	Interference filters	400 to 635	4	CCD	<2.0	Monitoring	Basal cell carcinoma, Bowen’s diseases	Skin	I	H	21
2016	Cust.	Spectral	Filter wheel	460 to 690	9 to 20	CMOS	0.13	Diagnosis	Melanoma	Skin	I	H	22
2017	Comm.	Spatial	FPI mosaic	600 to 1000	100	CMOS	<0.5	Imaging	Cancer	Skin	I	A	23
2017	Comm.	Spectral	LED	414 to 995	8	CCD	0.5	Diagnosis	Melanoma	Skin	I	H	24
2018	Cust.	Spectral	LED	995 to 1613	6	InGaAs	<1.0	Diagnosis	Melanoma	Skin	I	H	25
2018	Cust.	Spectral	LED	405 to 964	4	NA	<1.0	Diagnosis	Melanoma	Skin	I	H	26
2019	Cust.	Snapshot	LVF	400 to 700	9	CMOS	<0.5	Monitoring	Skin cancer	Skin	I	H	27
2020	Cust.	Spectral	LED	405 to 940	9	CMOS	<1.0	Monitoring	Skin cancer, erythema	Skin	I	H	28
2017	Cust.	Spectral	LED	448 to 659	3	CMOS	<0.5	Diagnosis	Nevi, hemangiomas, seborrheic keratosis	Skin	I	H	29
2011	Cust.	Snapshot	Mosaic filter	540 to 970	4	CMOS, CCD	0.1	Monitoring	Skin ulcer wounds	Skin	I	H	30
2017	Comm.	Snapshot	Holographic grating	600 to 1000	32	NA	0.36	Monitoring	Skin ulcer wounds	Skin	I	H	31
2012	Comm.	Spectral	AOTF	550 to 1000	NA	CCD	<1.0	Imaging	—	Skin	E	A	32
2017	Comm.	Spatial	Grating	500 to 980	100	CMOS	<1.5	Monitoring	Wound healing	Skin	I	H	33
2018	Comm.	Spatial	Transmission grating	500 to 1000	100	CMOS	0.45	Monitoring	Wound healing	Skin	I	H	34
2021	Comm.	Spatial	Transmission grating	500 to 1000	100	CMOS	0.45	Monitoring	Burn wounds	Skin	I	H	35
2021	Comm.	Spatial	Transmission grating	500 to 1000	100	CMOS	0.45	Monitoring	Wound healing	Skin	I	H	36
2018	Cust.	Spectral	LED	395 to 940	13	CMOS	<2.0	Diagnosis	Erythema	Skin	I	H	37
2019	Cust.	Spectral	LED	453 to 663	8	CMOS	<0.5	Diagnosis	Seborrheic dermatitis, psoriasis	Skin	I	H	38
2019	Comm.	Snapshot	SRDA	450 to 950	41	CCD	<0.5	Diagnosis	Necrosis	Skin	I	H	39
2019	Cust.	Snapshot	SRDA	470 to 630	16	CMOS	<0.5	Monitoring, diagnosis	Melanoma, vascular occlusion	Skin	I	H	40
2019	Comm.	Spectral	FPI interferometer	500 to 900	NA	CMOS	0.99	Diagnosis, monitoring	Vascular occlusion	Skin	I	H	41
2020	Cust.	Snapshot	Weiner estimation	482 to 506	16	CMOS	<0.5	Monitoring	Melanoma, vascular occlusion, CVD	Skin	I	H	42
2018	Comm.	Snapshot	Transmission grating	475 to 875	40	CMOS	0.36	Imaging	—		E	H	43
2013	Comm.	Spectral	Filter wheel	475 to 850	5	CCD	0.68	Detection	—	Veins	I	H	44
2012	Comm.	Spectral	AOTF	550 to 1000	NA	CCD	<1.0	Diagnosis	Head and neck cancer	Tongue	I	H	45
2013	Cust.	Snapshot	IMS	471 to 667	41	CCD	<1.0	Diagnosis	Head and neck cancer	Oral cavity	I	H	46
2020	Comm.	Spatial	SRDA	470 to 900	150+	CMOS	0.58	Diagnosis	Head and neck cancer	Thyroid, tongue, esophagus	E	H	47
2016	Comm.	Spatial	Grating	405 to 750	72	CMOS	NA	Diagnosis	Colorectal cancer	Colon	I, E	H	48
2019	Cust.	Spatial	LED	660 to 950	9	CMOS	NA	Diagnosis	Colorectal cancer	Colon	E	H	49
2018	Cust.	Spectral	SFDI	690 to 950	9	CMOS	<1.0	Diagnosis	Ovarian cancer	Ovaries	E	H, P	50
2016	Cust.	Spectral	LED	NA	10	CMOS	0.6	Diagnosis	Cervical cancer	Cervix	I	H	51,52
2019	Comm.	Snapshot	SRDA	450 to 950	41	CCD	<0.5	Diagnosis	Breast cancer	Breast tumor	E	H	53
2017	Comm.	Snapshot	Mosaic filter	460 to 630	16	CMOS	0.4	Diagnosis	Diabetic retinopathy	Retina	I	H	54
2017	Comm.	Snapshot	FPI mosaic	460 to 630	16	CMOS	<0.5	Diagnosis	Diabetic retinopathy	Retina	I	H	55
2016	Cust.	Spatial	Grating	480 to 705	NA	CCD	<1.0	Diagnosis	Alzheimer’s	Retina	I	A	56
2019	Comm.	Spatial	PGP	400 to 1000	467	CCD	1.1	Diagnosis	Alzheimer’s	Retina	I	H	57
2011	Comm.	Snapshot	CTIS	420 to 720	76	CMOS	<2.0	Diagnosis	AMD	Retina	I	H	58
2020	Cust.	Spectral	LED	405 to 700	9	CMOS	<0.5	Diagnosis	Chronic otitis media	Ear	E, I	P, H	59
2019	Comm.	Spatial	Transmission grating	400 to 1000	1004	CCD	0.68	Imaging	—		E	H	60
2011	Comm.	Spectral	LCTF	400 to 720	NA	CCD	0.375	Surgery	Tooth implant	Tooth	I	H	61
2016	Comm.	Spectral	LCTF	400 to 1700	NA	CCD, InGaAs	0.375	Surgery	Parotidectomy, colectomy, cystectomy	Facial nerve, colon, ureter.	I	H	62
2015	Comm.	Spectral	LCTF	400 to 720	NA	CCD	0.375	Surgery	Ischemia	Small bowel	I	H	63
2016	Comm.	Snapshot	FPI mosaic	481 to 632	16	CMOS	0.4	Surgery	Epilepsy	Cortex	I, E	H, P	64
2016	Cust.	Spectral	Spectral wheel	470 to 700	8	CCD	<2.0	Surgery	Colorectal cancer	Liver, gallbladder, colon, kidney	E	A	65
2019	Comm.	Spatial	PGP	400 to 1000	768	CMOS	1.8	Surgery	Colorectal cancer	Colon	E	H	66
	Comm.	Spatial	PGP	900 to 1700	240	InGaAs	4.4						
2010	Comm.	Spatial	PGP	400 to 1000	NA	CCD	1.1	Surgery	Ischemia	Intestine	I	H	67
	Comm.	Spatial	PGP	900 to 1700	NA	InGaAs	1.5						
2019	Comm.	Spatial	Transmission grating	500 to 1000	100	CMOS	0.45	Surgery	Colorectal resection	Colon	E	H	68
2018	Comm.	Spatial	Transmission grating	500 to 1000	100	CMOS	0.45	Surgery	Anastomotic insufficiency	Colon	E	H	69
2020	Cust.	Spatial	Transmission grating	500 to 1000	100	CMOS	0.26	Surgery	Head and neck cancer	Esophagus	E	P, H	70

Our literature search used the combination of the following search terms on three databases (Scopus, PubMed, and Web of Science): “compact” OR “miniature,” “hyperspectral” OR “multispectral,” and “camera” OR “imager.” We included only fully realized systems with definable dimensions and excluded developments in single components. There was a wide variety of contributions from industry, academic, and hobbyist space.

## 3 Historical Progress

The earliest applications of spectral imaging system were point scan cameras used for Earth remote sensing.^71^ A point scan camera called the Multispectral Scanner System (MSS) was used onboard the Earth Resources Technology Satellite (later called LANDSAT-1) in 1972.^2^ The system provided invaluable satellite images for the purpose of identifying, managing, and surveying geographical resources. To relay light, the system used a set of 24 fiber optic cables that transmitted light to the detectors. Because charge-coupled devices (CCDs) were not available at that time, the detectors used were photomultiplier tubes, which were extremely sensitive vacuum tubes that generated voltage upon radiation in the visible to near-infrared light range. Because of the extra components, MSSs were extremely bulky by today’s standards. The four-band system weighed up to 48 kg, measured 40×59×89  cm in dimensions, and consumed up to 42 W of power.^72^ Nevertheless, its success prompted NASA and Jet Propulsion Laboratory to develop multispectral cameras onboard future LANDSAT missions. In 1975, NASA launched LANDSAT-2 and equipped it with an even larger (64 kg, 54×62×126  cm) five-band MSS with added infrared capability. In 1979, NASA developed the airborne imaging spectrometer (AIS), which included a 32×32  pixels mercury cadmium telluride imaging sensor coupled with a silicon CCD multiplexer. AIS had a compact design to be flown on aircraft, measuring 30×30×20  cm in dimensions.^2^ A series of AIS followed, the most successful one being the airborne visible/infrared imaging spectrometer (AVIRIS). While new satellite-based spectral systems were being developed, the need for hyperspectral imagery in space-based applications was deemed unnecessary, and the only hyperspectral system that enjoyed long-term usage was the 202-wavelengths Hyperion camera onboard the Earth Observation-1 (EO-1).^2^ At the same time, commercial hyperspectral cameras for the purpose of land observation were developed with new imaging sensor technologies. An example was the compact airborne spectrographic imager (CASI), developed in 1989. CASI used a 512×288  pixels CCD to record up to 512 spatial pixels or 288 spectral bands in the 430 to 780 nm range.^73^

**Fig. 1:**
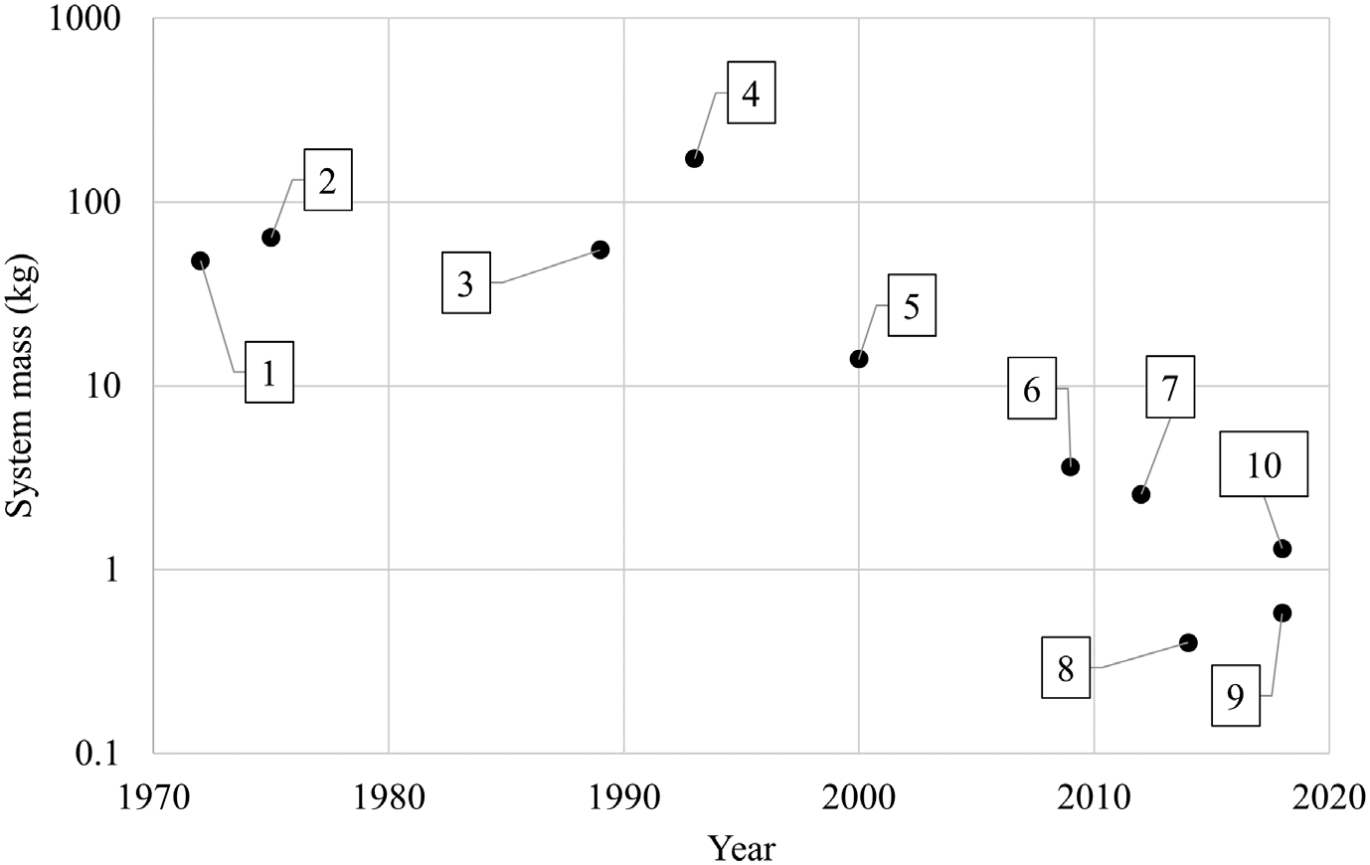
Timeline of the progress in size for spectral imaging. 1. MSS-1, 1972. 2. MSS-2, 1975. 3. CASI, 1989. 4. Digital airborne imaging spectrometer, 1993. 5. Compact high-resolution imaging spectrometer, 2000. 6. Headwall Hyperspec, 2009. 7. Resonon PikaL, 2012. 8. BaySpec OCI-1000, 2014. 9. Imec Snapscan, 2018. 10. Specim IQ, 2018. After the 2000s, spectral imaging systems became remarkably smaller due to manufacturing advances. All selected imaging systems required motion of the camera or the sensor to acquire the spectral cube.

**Fig. 2:**
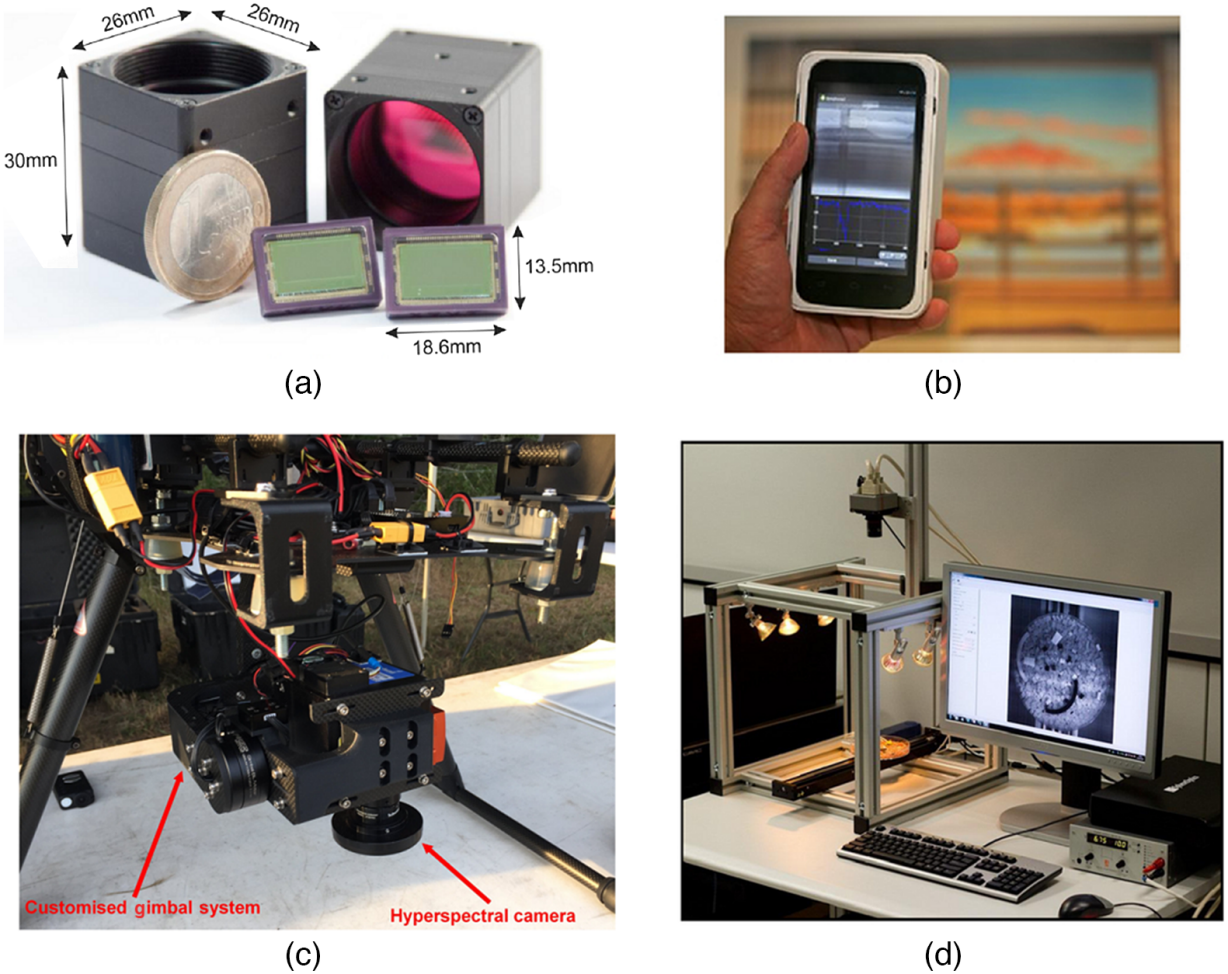
A selection of ultracompact spectral imagers. (a) Two snapshot imaging cameras that weighed <30  g (reproduced from Ref. 76). (b) A handheld snapshot camera that weighed <500  g (reproduced from Ref. 77). (c) A spatial scanning camera used in UAV applications (reproduced from Ref. 78). (d) A snapscan imaging camera being used in a laboratory setting (reproduced from Ref. 79).

Up until 2000, progress in spectral imaging left much to be desired, especially when compared with parallel progress in commercial cameras and other consumer electronics.^74^ Developments of compact spectral imaging systems prospered after the 2000s, due to developments of portable spectroscopy.^8^ These developments did not stem from any new optical architecture, but rather manufacturing methods that matured enough to create miniature imaging components. The number of transistors in an integrated circuit has been observed to double every two years for the last 50 years, this is colloquially known as Moore’s law. Smaller imaging sensors that followed Moore’s law, lithography processes, microelectro-mechanical systems (MEMS), microcontrollers, and 3D printers were some innovations that made new spectral cameras smaller and lightweight. With advances in unmanned aerial vehicles (UAVs or drones) for consumers, ultracompact spectral imaging systems were being developed for the purpose of being carried by UAV.^75^ Modern UAVs have payload limits ranging from 125 g to 5.5 kg, limiting the maximum weight of compact spectral cameras.^76^ However, UAVs were not the only impetus for compact and ultracompact imaging systems. As other fields, such as biomedical imaging, industrial imaging, and environmental monitoring, moved from laboratory analysis to on-site imaging, the demand for compact spectral cameras grew larger. In the early phase of developments, tradeoffs were often required between performance and size. However, new generations of ultracompact devices seemed to overcome those limitations altogether, as demonstrated by a recent line scan imaging system that weighed only 400 g (∼0.9  lbs), yet was capable of capturing ∼100 spectral bands at a resolution of <5  nm.^77^ Figures 1 and 2 show a sample of spectral cameras over time to show the progress in size.

## 4 Acquisition Overview

**Fig. 3:**
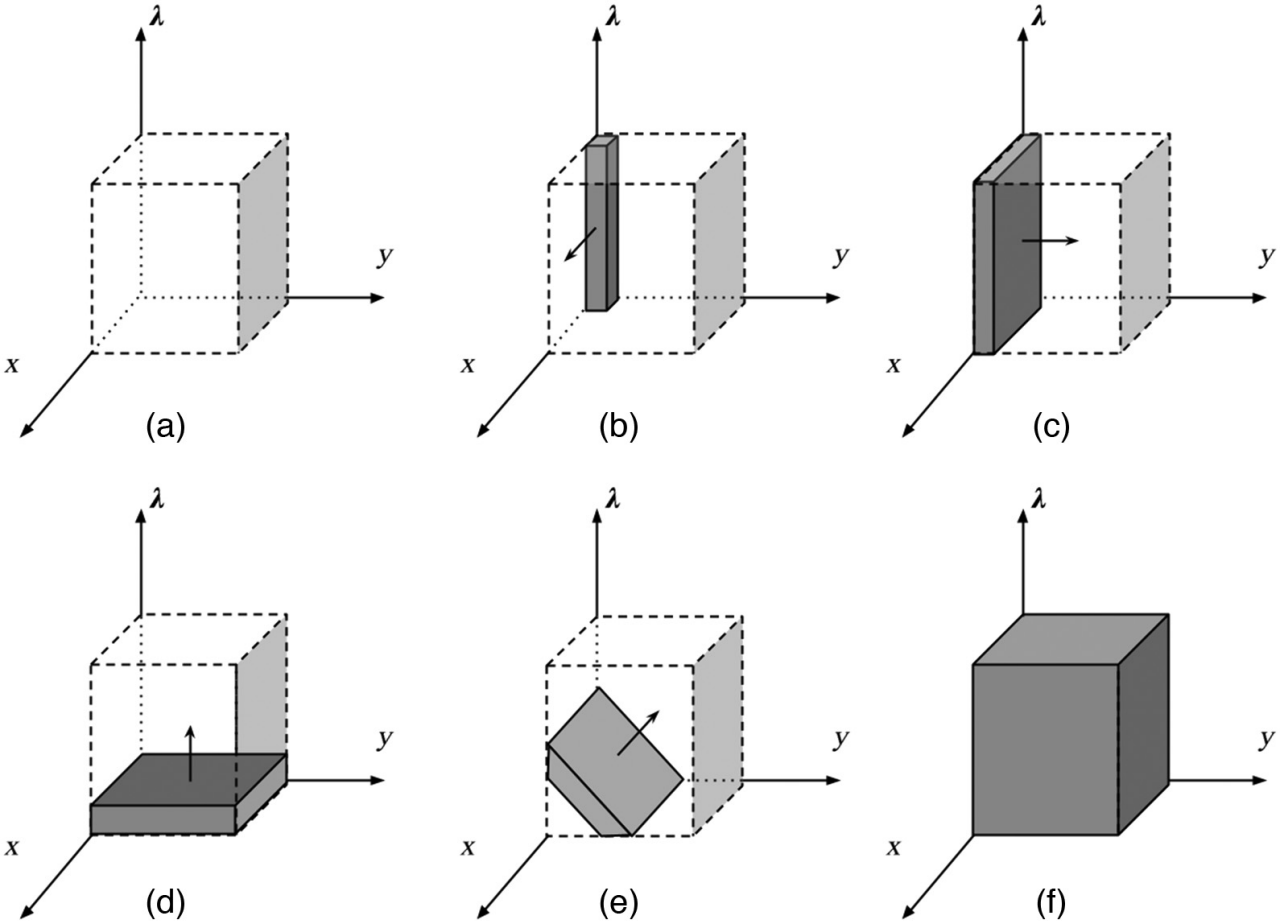
Comparison of different methods of datacube acquisitions. The shaded regions correspond to the section captured in one exposure. The arrows show the direction of scanning. (a) The hypercube. (b) Point scanning. (c) Line scanning. (d) Spectral scanning. (e) Spatial–spectral scanning. (f) Snapshot scanning.

The goal of spectral imaging is to acquire a datacube, a 3D block of data with two spatial dimensions and one spectral dimension (Fig. 3). In a datacube, the unit of smallest resolution is called the voxel, which is the equivalent of a pixel in digital images. The spatial dimension represents the field-of-field of interest, and the spectral dimension represents the different wavelengths. There are five methods of acquiring the datacube based on how much of the datacube is being captured within one exposure: point scanning, line scanning, spatial–spectral scanning, spectral scanning, and snapshot imaging.^80^ Because spatial–spectral scanning relies on translation to image the entire data cube, we grouped spatial–spectral scanning as a subset of line scanning.^81^ Acquisition methods can also be classified based on how they acquire the spectral component: either through interference filters, monochromators, or interferometers.^80^ Both interferometers and interference filters use the same optical mechanisms. They both consists of devices that superimposed light and produced interference patterns. However, interference filters typically use interference to block certain amount of light, whereas interferometers use interference to generate signals that can be measured and processed down the line. Each method offers their own engineering strengths and weaknesses, so choosing an appropriate method for datacube acquisition for specific applications is important.

### 4.1 Point Scanning and Line Scanning

Point scanning and line scanning, collectively called the spatial scanning or imaging spectrograph, acquires the entire spectrum section-by-section and uses mechanical means to scan the entire space. These methods are also known as “whiskbroom”/ “spotlight” and “push-broom,” respectively, which are terminologies originated from satellite imaging. In a point scanning imager, an aperture only allows light from a small section to pass through a monochromator, which will disperse the light onto a sensor array. The specific method of scanning point by point in whiskbroom imagers brings to mind that of confocal microscopy imaging, in which a pinhole is also used to block lights that are out of focus. Hyperspectral confocal microscopy combines both methods with little modifications, producing 3D structures that can be analyzed by spectral values.^82^^,^^83^ In a line scanning imager, the aperture is a slit that allows a sliver of light to be dispersed onto a two-dimensional (2D) sensor plane (the direction of dispersion is perpendicular to the direction of the slit). Compared with point scanning, line scanning can capture the same field of view in less time, but the higher number of sensors means that there are potentially more elements to calibrate and higher chance of sensors artifact.^6^ Line scanning remains the dominant method of acquisition in biomedical imaging^7^ and remote sensing.^81^

As mentioned, usage of spectral imaging for practical purposes began with the MSS in 1972. In this system, the point-scanning imager used a mirror and the motion of the spacecraft to achieve perpendicular and parallel scanning, respectively.^84^ Nowadays, in the field of remote sensing, mirrors combined with motion of the aircrafts are still being used as the mean of acquiring the full spatial component.^71^^,^^85^ By contrast, in histology, fluorescence, and confocal microscopy imaging, the subject being imaged is moved with the help of programmable platforms.^60^ Several line scanning cameras that used mirrors to image near objects were proposed. However, these devices all required spatial calibration to avoid image distortions.^86^^,^^87^ In the works by Sigernes et al.^86^ and Gutiérrez-Gutiérrez et al.,^87^ the mirror is on a rotational axis parallel to the line scanning slit. When the mirror rotates, only a line section gets reflected and focused onto the dispersive device.

### 4.2 Spectral Scanning

In spectral scanning (also called staring, framing, or band sequential), the datacube is captured spatially all at once but only at selected wavelengths. The process of wavelength selection is done using bandpass spectral filters, which can be either interference filters, variable filters, or interferometers. Because no filter has an infinitely small bandwidth, the resulting image captured at each wavelength should be considered more as a function of the filter’s spectral response, quantified by the following equation:

$$I_{\left(i\right)}=\int_{0}^{\infty }m_{i}(\lambda )r(\lambda )d\lambda .$$

In this equation, $I_{\left(i\right)}$ is the intensity of the datacube captured by filter i, $m_{i}(\lambda )$ is the spectral response of the filter, and r(λ) is the aggregated spectrum that reaches the imager. Some early spectral scanning systems used filter wheels, which were interference filters that can be switched in and out. As the name suggests, the filters were arranged in a circle. During capture, the entire wheel rotated and cycled through every waveband. The advantages of filter wheels included cost and simplicity. However, the disadvantages of filter wheels included speed, size, lack of customization, and small number of bands. An alternative to mechanical filter wheels was electronic tunable filters, which were mainly acoustooptical tunable filters (AOTF),^88^^–^^90^ liquid crystal tunable filters (LCTF),^62^^,^^63^^,^^91^^–^^93^ or Fabry–Perot interferometers (FPI). There existed other mechanisms for manufacturing tunable filters, such as surface plasmon coupled tunable filters;^94^ however, three types described earlier are still the most popular. LCTF uses birefringence crystals to bandpass light. In LCTF, birefringent crystals and polarizing filters are stacked in alternating layers. Modifying the birefringent crystal’s retardance with electrical inputs results in different polarizing states of the output wavelength and in turn bandpass only selected wavelengths.^91^ AOTF uses crystal that selectively bandpass wavelengths based on the radio frequency inputs. AOTF functions similar to a diffraction grating but only has one constructive waveband passing through.^95^ Compared with LCTF, AOTF have faster switching time and less power demand.^93^ They also have no moving component, making them the preferred method for vibration-sensitive applications.^93^ Interferometers refer to a broad class of spectral imaging layouts that use different reflective surfaces layouts to produce interference, such as the Michelson interferometer, the Sagnac interferometer, the Mach–Zehnder interferometer, and the FPI.^96^

### 4.3 Spatial-Spectral Scanning

Spatial–spectral scanning (also called windowing) captures the datacube in both the spatial and spectral directions within one single exposure and effectively samples a diagonal “slice” out of the datacube. Even though the datacube reconstruction is harder to visualize in spatial–spectral scanning, this method offers advantages in acquisition speed and movements. Methods of spatial–spectral scanning often used dispersion elements that were location-dependent, such as linear variable filters (LVFs).^97^ With the use of a simple grating element in the fore optics, a line scanning spectral camera can be converted into a spatial–spectral scanning camera.^98^ The grating element transmitted lights that pass through the aperture slit based on both spatial and spectral values. Pichette et al.^79^ developed a commercial device called the “Snapscan” imager that used location-dependent interference filters. In their system, a series of Fabry–Perot filters overlaid the imaging sensor. The bandpass values of the filters varied across the translation direction, so by moving the sensor both spatial and spectral components were sampled. Fourier transform spectrometer can also be used as a mechanism to achieve spatial–spectral scanning.^13^^,^^99^

### 4.4 Snapshot Scanning

Snapshot scanning cameras capture the entire spatial structure multiple wavelengths at one exposure.^100^ The implementations of snapshot cameras are extremely diverse and can be classified into the following main categories: dispersive-based methods, coded-aperture methods, speckle-based methods, and spectral-resolved detector methods.^101^ Dispersive-based methods rely on dispersive elements (grating or prisms) to split the incoming images into different wavelengths that are recorded either on a single sensor or on multiple sensors. An example of a system that uses multiple sensors to record the spectral images is the beam splitting camera.^100^ These types of cameras are often seen in television and movies production, although they only have three sensors for red, green, and blue images. An example of a system that records the entire datacube on a single sensor is the computed tomographic imaging spectrometry (CTIS). CTIS is popular in the hobbyist and academic space, as it is easy to construct with low-cost components.^102^^–^^104^ However, the reconstruction of the datacube using CTIS requires using inverse projection, which can be computational-consuming. Another problem is that CTIS records both the spatial and spectral data on a single sensor, which requires a tradeoff between spatial and spectral resolution. Coded-aperture methods use a patterned filter (mask/coded aperture) at the location of the aperture, which will “encode” the incoming wavelengths by lossy compression. The compressed data can record more light compared with CTIS, but this also means that some of the wavelengths will be lost.^105^ Speckle-based systems reconstruct the datacube through correlating the speckle data and the wavelengths. Spectrally resolved detector arrays (SRDA)^39^^,^^53^^–^^55^ use interference filters manufactured on top of imaging sensor to capture snapshot images. For example, color filter arrays, the most popular of which are Bayer filters, are used in consumer digital cameras to simulate human color vision.^106^ Snapshot cameras that use SRDA are fast, compact, lightweight, and require no additional movement mechanics. However, they downsample the spatial dimension, which can result in aliasing of the spatial data if the Nyquist limit is not obeyed. There is also a tradeoff between the number of filters and the spatial pixel resolution: the higher the number of different filters are, the lower the light throughput.^100^ Readers who are interested in snapshot imaging implementations should consult a literature review by Hagen and Kudenov^100^

### 4.5 Conversion from RGB to Spectral Images

There existed a separate line of research that generates spectral images without using spectral cameras. Regular color vision cameras use three color filters in the red, blue, and green (RGB) wavelengths to simulate human color vision. However, these filters are broadband filters with a large amount of overlap, and transformation algorithms can be used to extrapolate multispectral or hyperspectral data. Furthermore, hyperspectral data are often sparse, making recovery of spectral data more feasible.^107^ All conversion methods generated a mapping function that transform three-wavelengths data to multiwavelengths data. The function was approximated by minimizing the differences between generated spectral data and expected spectral images. One of the first methods used for this was Wiener estimation.^42^^,^^108^^,^^109^ Arad and Ben-Shahar^107^ used match pursuit algorithm to construct a mapping from natural RGB to hyperspectral images. In recent years, machine learning algorithms, including neural networks, were investigated to learn the mapping. Koundinya et al.^110^ proposed 2D and 3D convolutional neural networks (CNN) systems that mapped RGB images to 31-waveband images in the wavelengths 400 to 700 nm. Alvarez-Gila et al.^111^ used generative adversarial networks (GAN) to produce spectral images from RGB. All these methods still faced significant constraints. As noted by Signoroni et al.^112^ in a review of neural networks for spectral images analysis, several of these generation methods could only generate outputs in the visible light range. Furthermore, they required extensive training inputs of RGB images and corresponding spectral images, which by itself needs a spectral camera to capture. Most input images used for training were outdoor images, which can make the mapping unsuitable for indoor or laboratory settings.

### 4.6 Comparisons of Acquisition Methods

The rule of thumb has been that spatial scanning cameras can achieve high spatial and spectral resolutions at the cost of acquisition time, snapshot cameras are fast, and spectral cameras achieve high spatial resolution while trading between acquisition time and spectral resolution.^6^ Considering the state-of-the-art available, it is important to re-examine this convention. We start by comparing two key spectral variables: the spectral resolution (defined as the full-width half-maximum or FWHM value at center wavelength^8^) and the spectral range. In this paper, we use the term “bandwidth” interchangeably with FWHM. Some authors such as Hagen and Kudenov^100^ found the term “resolution” in digital spectral cameras to be confusing as it can also be used to describe the number of spectral samples. Point scan and line scan systems have reliable spectral resolution: many commercial systems have FWHM in the range of 2.5 to 5 nm and are capable of capturing more than 100 spectral bands at once.^81^ One line scan system for remote sensing reported FWHM as narrow as 1.85 nm.^113^ Spectral scanning systems that use filter wheels typically achieved 3 to 10 bands^38^^,^^114^^–^^116^ and each individual filter had bandwidth ranging anywhere from 30 to 250 nm.^117^ On the other hand, tunable filters can achieve very fine spectral resolution. AOTF could achieve a bandwidth of 0.1 nm;^118^ however, commercially available AOTF filters often had a minimum FWHM in the 2 to 6 nm range.^91^^,^^119^ LCTF systems often had larger spectral resolution compared with AOTF, ranging from 4 to 30 nm.^91^ Interferometers typically achieve good spectral resolution: FPI that were driven by piezoelectric had spectral bandwidth in the range of 10 to 20 nm.^120^ The FWHM also affects the light throughput, as interference filters with smaller bandwidths have lower light throughput. Some filters’ specification uses the term “optical density” to quantify the amount of energy blocked. Applications that need low signal such as autofluorescence imaging require an optical density of 6 or greater. However, low signal also means low signal-to-noise ratio (SNR) and affects the image quality.

In terms of functional ranges, the wavelength ranges of these devices were more dependent on the sensor types than the acquisition method. The functional range of silicon-based sensors is constrained in the range 400 to 1000 nm because of silicon’s photoelectric properties. If researchers wanted to investigate wavelengths in the short-wave infrared (SWIR) and higher (1000 to 12,000 nm), an alternative semiconductor material such as indium gallium arsenide (InGaAs, also named GaInAs) should be used. In terms of spectral wavelength selection, LCTF, AOTF, and FPIs provide an advantage over spatial scanning, snapshot scanning, and filter wheels. That is because the former can tune to arbitrary wavelengths while the latter have discrete wavelength selection. In many cases, specific selection of center wavelengths was not a major concern, so this advantage was not often used.

The spatial pixel resolution in spatial scanning and spectral scanning system is dependent mostly on the sensor resolution and the binning (grouping of several pixels into one) used. In snapshot cameras, the optic architecture determines the maximum pixel resolution available. If the snapshot architecture captures the entire datacube representation upon a single detector plane as in the case of CTIS, IMS, and SRDA architectures, then the spatial resolution is often poor. Ford et al.^12^ used a 2048×2048  pixels detector to capture a datacube of dimension 203×203×55 using CTIS. A recent CTIS system captured up to 315 wavebands but has a pixel resolution of 116×110  pixels.^102^ Many other commercial snapshot cameras could capture up to 100 or more wavebands but had spatial pixel resolution of not more than 100×100.^81^ The acquisition time of point scanning, line scanning, and spatial–spectral scanning systems can be described as rapid. A recent series of line scanning cameras to be used in industrial settings were capable of scanning 576  spatial line/s or 2880 spatial–spectral elements/s.^121^ Because spatial and spatial–spectral scanning systems are used in many industrial and remote sensing environments, the readout speed of these systems also depends on the translation speed of the camera or the samples. The acquisition time of spatial scanning systems relies on the switching time of the filters. Filter wheels achieve switching time in orders of seconds, LCTF in orders of 50 to 500 ms, and AOTF in orders of 10 to 50 *μ*s.^93^^,^^100^ As for FPI, the switching time ranges from 5 to 50 ms,^122^ although <3  ms switching time had been reported.^123^ The fact that snapshot cameras can capture the datacube in one exposure does not necessarily mean that they have low acquisition time. Snapshot systems using mosaic filters can be very fast: one system was capable of capturing a 256×256×32  pixels datacube at a rate of 340 images per second.^121^ The processing time was part of the consideration for a very long time. Unlike spatial scanning, spectral and snapshot scanning methods all required postprocessing to stitch together the spectrum.^74^ Some methods required extensive processing to generate the datacube, such as CTIS (Fourier slice theorem) or Fourier transform spectroscopy (inverse Fourier transform). However, with advances in computer power, the processing time after acquisition to generate the datacube is becoming more similar across all platforms.^100^

Light throughput, the amount of light in the datacube that the detector can measure, affects the SNR. Theoretically, it is been long known that some methods of spectroscopy acquisition have higher light throughput compared with others, which in turn increases the SNR.^100^ However, among spatial and spatial-spectral scanning systems, the theoretical differences in SNR are not significant.^14^ Hagen et al.^124^ argued for the snapshot advantage, which is the increased throughput that comes from the fact that snapshot cameras capture the entire datacube at once. However, the snapshot advantage is only available for a select number of snapshot architectures, such as CTIS, IMS, and CASSI. Realistically, throughput also depends on the filter transmission rate and the quantum efficiency (QE) of the sensors. Finally, some imaging applications are more suitable for certain methods of acquisitions. Confocal microscopy, for example, can only be coupled with point scanning imaging due to the fact that only a small section of light is imaged.^100^

## 5 Technical Aspects of Compact Spectral Cameras

Components of a spectral imager include the optical system (such as lens, endoscope, microscope), the spectral dispersion system (such as monochromator or interferometer), the digital image detector, the control module, and the mechanical elements (gears and housing).^6^ As spectral and optical systems are dependent on how the datacube is acquired, we decided to group them by acquisition methods instead. For some spectral imaging systems, illumination is also a critical component.

### 5.1 Spatial Scanning

**Fig. 4:**
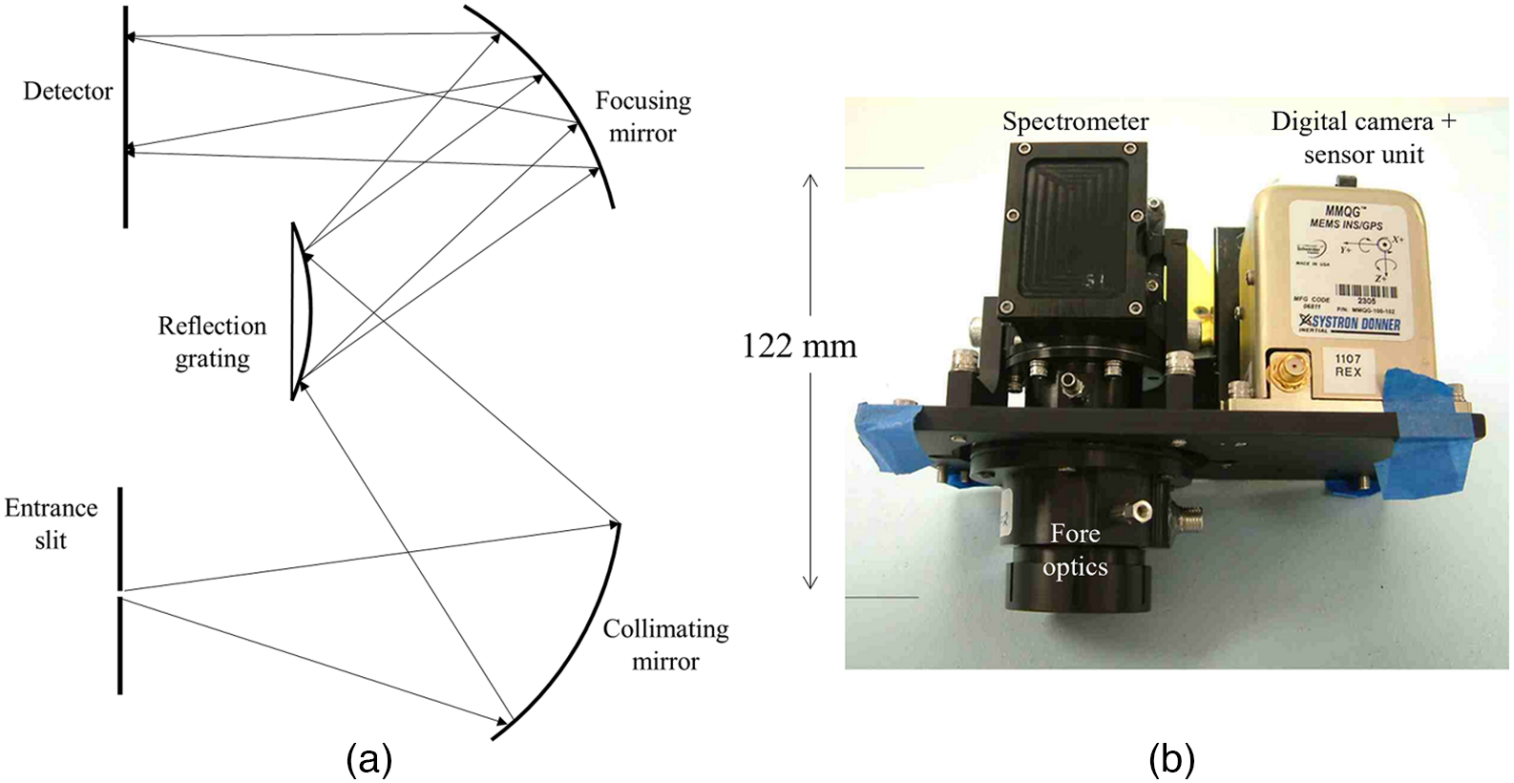
Offner spectrograph is an example of a reflective grating configuration. (a) The working mechanism of an Offner spectrograph camera (adapted from Ref. 125). (b) An example of a device that uses Offner spectrograph (reproduced from Ref. 126).

Miniature spatial scanning systems use two main classes of architectures: reflective grating and transmission grating/prism.^74^ In both systems, after the light passes through the aperture, it gets collimated (made parallel), then dispersed, and then the individual wavelengths will be refocused onto the array detector.^15^ In the Czerny–Turner reflective grating configuration, a curved mirror acts as the collimating mirror and a second curved mirror refocuses the diffracted light onto the imaging sensor. In the Offner configuration, three concentric elements (collimating mirror, reflective grating mirror, and focusing mirror) make up the optical components [Fig. 4(a)]. Even though the Offner configuration provides better spectrographic ability, manufacturing them was not possible until the 1990s due to the lack of precision lithography technology.^74^ Offner spectrometers are commonly used due to their low aberration.^127^ Warren et al.^126^ used a monolithic block of glass as the transmitting medium for the Offner relay, reducing the volume and weight of the imager down to 0.54 kg [Fig. 4(b)].

**Fig. 5:**
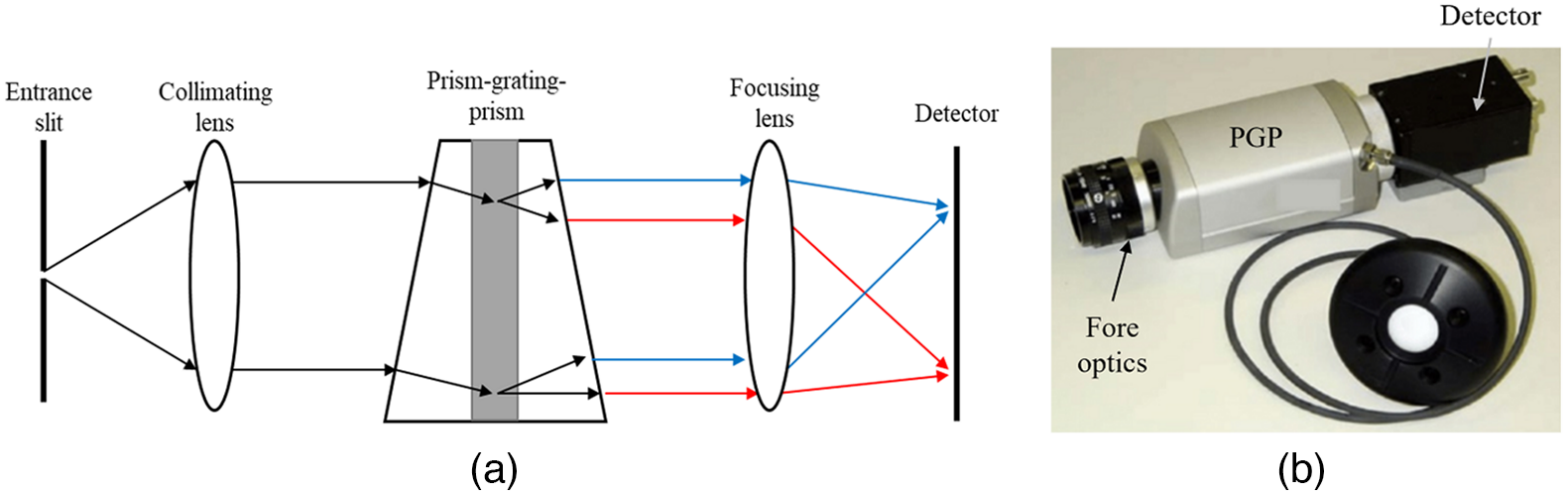
PGP is an example of a transmission grating configuration, where no reflective device was used. (a) The working mechanism of a PGP imaging camera (adapted from Ref. 125). (b) An example of a device that uses PGP (reproduced from Ref. 138).

Many low-cost and compact systems used prisms^128^^–^^130^ or transmission grating^70^^,^^85^^,^^102^^,^^131^^–^^133^ as the monochromator. The problem was that these systems were prone to artifacts due to misalignments of the optical components.^85^ Some key artifacts included chromatic aberration (lens not focusing all wavelengths properly), smile (bending of the spectral line), keystone (bending of the spatial data), and straylight (unwanted light caused by other sources).^134^ Some optical architecture showed advantages in producing fewer artifacts. For example, Offner spectrometers have less smiles and keystones compared with Czerny–Turner and transmission grating architectures.^10^^,^^135^ High quality optical components, such as achromatic lenses, also reduce aberration to a degree, but they can potentially increase the manufacturing cost of the device. Laboratory calibration can also be done to reduce smile and keystone.^127^ In commercial spatial scanning systems, smile ranges between −0.1 and 0.1 pixels and keystone reaches a maximum of 3.5 pixels at 1000 nm.^136^ Grism (prism and grating) can reduce some chromatic aberration and is also a common monochromator used in some compact spectral imagers.^134^^,^^137^ Prism–grating–prism (PGP) was first seen in the works of Aikio^74^ as a method of building ultracompact push broom imagers. In a PGP, two identical prisms sandwich a volume transmission grating [Fig. 5(a)]. Compared with prism and transmission grating alone, PGP disperses light linearly, has high throughput, and is extremely robust.^74^ The greatest advantage of PGP, however, is the ability to disperse light with little space, allowing the development of miniaturized spectral imaging systems. Multiple compact spectral imagers used PGP as their dispersive element^121^^,^^137^^,^^139^^–^^141^ [Fig. 5(b)]. Some commercial systems also use high quality manufactured transmission holographic grating, such as the one reported by Wu et al.,^77^ which demonstrated an ultracompact line scan imager using volume phase grating. Table 2 summarizes the different common methods of dispersion used in spatial scanning imaging cameras.

**Table 2:** Optical comparison among common types of spatial scanning imagers.

Type	Collimating method	Dispersion method	Focusing method
Czerny–Turner spectrometer	Concave mirror	Flat reflective grating	Concave mirrors
Offner spectrometer	Concave mirror	Convex reflective grating	Concave mirrors
Prism–grating–prism	Optical lenses	A transmission grating between two prisms	Optical lenses
Holographic grating	Optical lenses	Volume phase holographic transmission grating	Optical lenses
Grism	Optical lenses	Transmission grating followed by prism	Optical lenses

Due to their nature of acquisition, many laboratory-based spatial scanning imagers require either the stage to move or the camera to move. These methods of acquisition are not suitable for bioengineering applications such as surgical guidance or *in vivo* imaging.^129^ To develop spectral imagers that can be compact and usable for live imaging, new techniques in spatial scanning acquisition have been devised to overcome the movement problem. These techniques use microelectronic internal devices such as digital micromirror devices (DMD)^142^ and piezoelectric motors^70^^,^^79^^,^^143^ to move the imaging sensors or optical components. Several commercial systems, such as those shown by Wu et al.^77^ and Behmann et al.,^144^ are fast enough that they can capture accurate spatial data entirely handheld.

### 5.2 Spectral Scanning

**Fig. 6:**
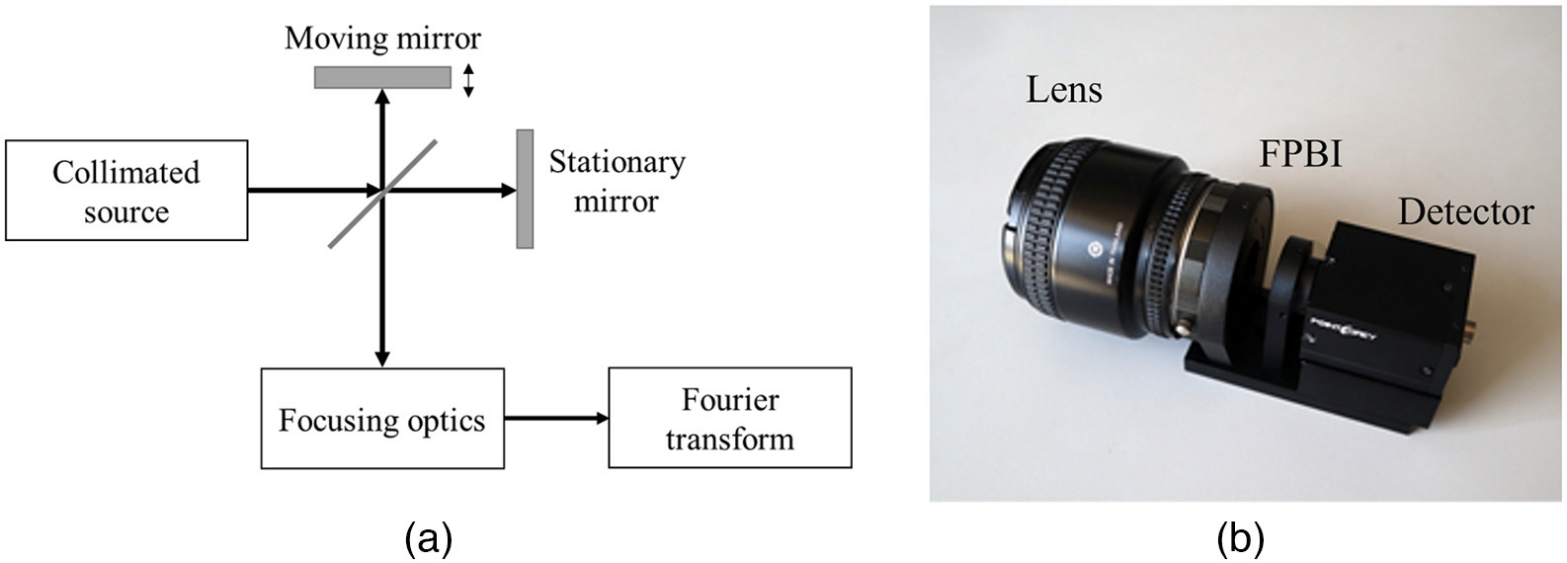
FTIS. (a) Working mechanism of an FTIS system (adapted from Ref. 148). (b) Image of a compact FTIS camera that uses a focal plane birefringent interferometer (FPBI in the figure) in front of the detector (reproduced from Ref. 149).

**Fig. 7:**
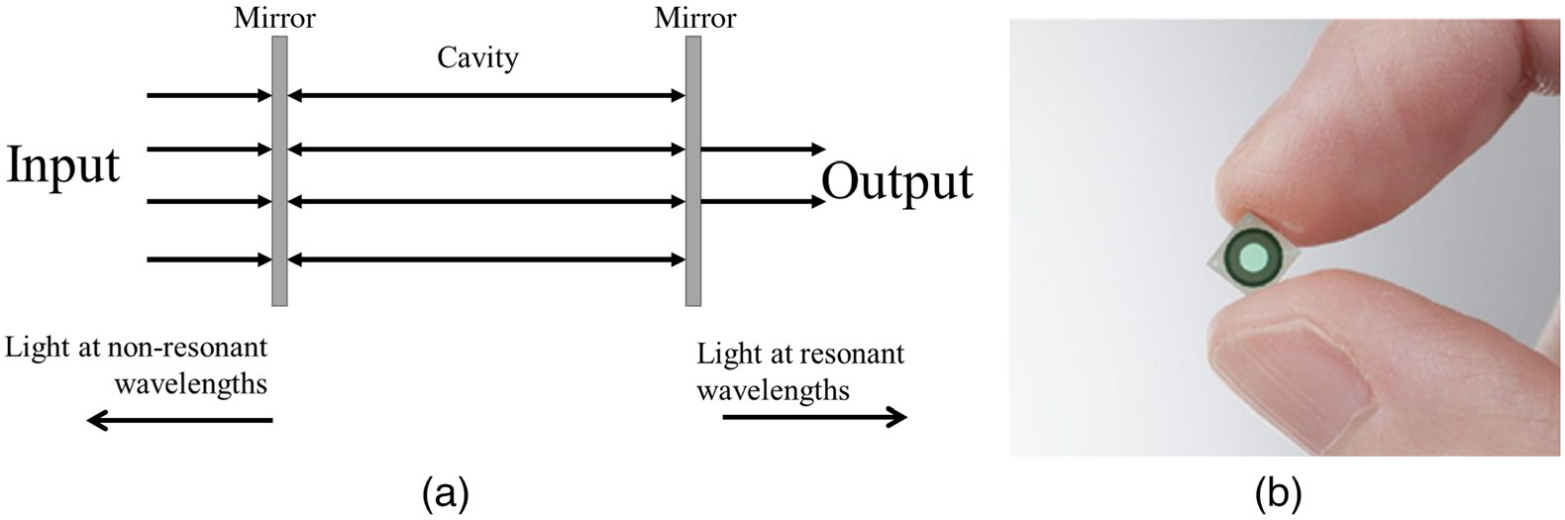
FPI. (a) Working mechanism of an FPI system (adapted from Ref. 150). (b) A compact FPI chip that is driven by electrostatic actuation (reproduced from Ref. 151).

Of the compact spectral scanning systems that we surveyed, tunable filters were preferred over filter wheels due to their small size and narrow bandwidth. Some have succeeded in miniaturizing filter wheel systems. For example, Kim et al.^22^ applied a filter wheel with nine wavebands on top of a smartphone camera. While electrically tunable filters can achieve arbitrary waveband selection, efforts to miniaturize them were hindered by the lack of suitable compact configurations. Both AOTF and LCTF require large external power and large optical pathway, which makes them unsuitable candidates for ultracompact systems.^94^ While the size of the filters in AOTF and LCTF themselves is compact and lightweight (often <1  kg),^88^^,^^89^^,^^118^^,^^145^^,^^146^ it is the weight of filter driver that adds up to the weight of these devices. Ishida et al.^147^ were able to deploy an LCTF-based system on top of a UAV. However, the UAV was only able to fly for 10 min due to the high payload weight. On the other hand, interferometric imaging spectrometers are becoming increasingly compact and have found their way into many applications. We discussed two primary types of compact spectral cameras that use interferometers as their dispersive elements: Fourier-transform imaging spectrometers (FTIS) and FPIs (Figs. 6 and 7). All the previously discussed systems used filters to filter out broadband light sources. However, the light sources themselves can be used as mechanisms for spectral scanning. In such systems, there is an arrangement of narrow-band light sources that illuminate the subjects at different wavelengths. The reflected light measured by the electronics detector is analogous to the spectral response of the subjects. Light-emitting diodes (LEDs) are a common method to achieve variable lighting useful for multispectral or hyperspectral systems. Various LEDs are discussed in Sec. 5.6.1. Table 3 compares the common methods used to produce spectral scanning imagers.

**Table 3:** Comparisons of several types of spectral scanning cameras. AOTF, acousto-optic tunable filter; LCTF, liquid crystal tunable filter; FTIS, Fourier transform imaging spectrometer; FPI, Fabry–Perot interferometer; FWHM, full-width at half-maximum (measured in nm). Wavelengths refer to the operating range of wavelengths. VIS, visible wavelengths (400 to 700 nm); NIR, near-infrared wavelengths (700 to 2000 nm). Switching speed refers to time to change from one wavelength to another one; ms stands for milliseconds (1/1000 s).

Type	Wavelengths operating range	FWHM (nm)	Number of bands	Switching speed
Filter wheel	VIS-NIR	30 to 250	<50	∼1 s
LED-based	VIS	20 to 70	<100	∼500 ms
AOTF	VIS-NIR	2 to 6	>1000	∼1 ms
LCTF	VIS-NIR	5 to 30	>1000	∼100 ms
FTIS	VIS-NIR	10 to 50	>1000	∼10 ms
FPI	VIS-NIR	10 to 50	>1000	∼10 ms

#### 5.2.1 Fourier transform imaging spectrometers

An interferometer splits the incoming light wave into two light waves that are then superimposed onto each other. The superimposition has a slight delay, causing a wave pattern (interferogram) whose magnitude is dependent on the delay. In the early 19th century, Albert Michelson showed that one can use Fourier inverse transform to convert the interferogram into actual spectra of the incoming light.^152^ This was the basis of Fourier transform spectrometry [Fig. 6(a)]. When the detector noise dominates other sources, Fourier transform spectrometry has lower SNR compared with dispersive-based spectrometers and higher throughput compared with slit-based spectrometers.^153^ However, previous imaging spectrometers that used mechanical interferometers (also called Michelson interferometers) suffered from many drawbacks. Mainly, they required accurate mechanical movement, making them unsuitable for field deployment.^154^ In recent years, birefringent (different refractive index based on light polarization and propagation) crystals were being used to generate compact interferometers in FTIS.^96^^,^^149^^,^^155^ The majority of birefringent crystal schemes were either Wollaston or Savart prisms.^155^ Both optical schemes used prisms to separate polarized light and collimate them to introduce a delay in the light. Perri et al.^155^ introduced a birefringent interferometer called translating-wedge-based identical pulses encoding system and commercialized a compact hyperspectral camera using this system. Xu et al.^149^ used a birefringent interferometer at the focal plane to produce an ultracompact spectral camera [Fig. 6(b)].

#### 5.2.2 Fabry–Perot interferometers

In its simplest form, an FPI is an arrangement of two parallel or curved mirrors that bandpass wavelength based on the separation distance between the mirrors. Suppose two highly reflective surfaces are separated by a distance d apart by a separation medium with refractive index n, then a collimated beam arriving at normal angle will exhibit transmittance by constructive interference at the wavelengths 2dn=mλ for m=1,2,3…^156^ with all other wavelengths being almost entirely reflected by the Fabry–Perot filters [Fig. 7(a)]. An FPI could then become a tunable filter by varying the distance between the two mirrors. However, FPI filters allow transmission of wavelengths that are periodic, and the distance between the two wavelengths that are transmitted is called the filter-free spectral range (FSR):

$$\mathrm{FSR}=\frac{\lambda^{2}}{2d}.$$

In this equation, the FSR and the distance between the plates are inversely related. This means that if we want to increase the range of the FPI, a low value of d needs to be selected. If the FPI operates within the infrared region, this value could be as low as several microns.^157^ Like other FTIS, FPI has a throughput advantage compared with dispersive-based spatial scanning cameras. However, the realization of FPI in spectrometry was very recent since their fabrication required highly reflective surfaces to decrease the spectral resolution. Instead of a singular reflective medium, alternating high and low refractive index materials were arranged to create highly reflective Bragg mirrors.^158^^,^^159^ The reflectivity of the materials affects the FWHM value of the filter in the following relationship:

$$F=\frac{\pi }{\sqrt{R}(1-R)},$$

where F is the FWHM and R is the reflectivity of the cavity mirrors. If the distance between the mirrors is unchanged, FPI is also called etalon and is more often used in LVFs and snapshot scanning cameras (see Secs. 5.3.2 and 5.3.5). There are many methods to vary the distances between the mirrors. In piezo-actuated methods, piezo devices produce strong physical displacements when voltage is applied. In capacitive or electrostatic actuated methods, the moving plate is tensioned by springs and moves to electrostatic force^157^ [Fig. 7(b)]. FPI can be manufactured through photolithography and assembled either through surface micromachining or bulk micromachining (see Sec. 5.4 for more discussions). This process of manufacturing enables ultracompact FPI filters. From the equations, it is important to notice that the constructive interference in FPI not only allows the central wavelength to pass through but also other secondary wavelengths that are multiples of the central wavelength. Appropriate long-pass and short-pass optical filters should be used.^54^

### 5.3 Snapshot Scanning

The mechanisms used to acquire a spectral cube in a snapshot manner are numerous. Here, we describe the common methods used in compact spectral imaging, which were CTIS, SRDA, compressive sensing, image mapping spectrometer (IMS), and LVF.

#### 5.3.1 Computed tomographic imaging spectroscopy

**Fig. 8:**
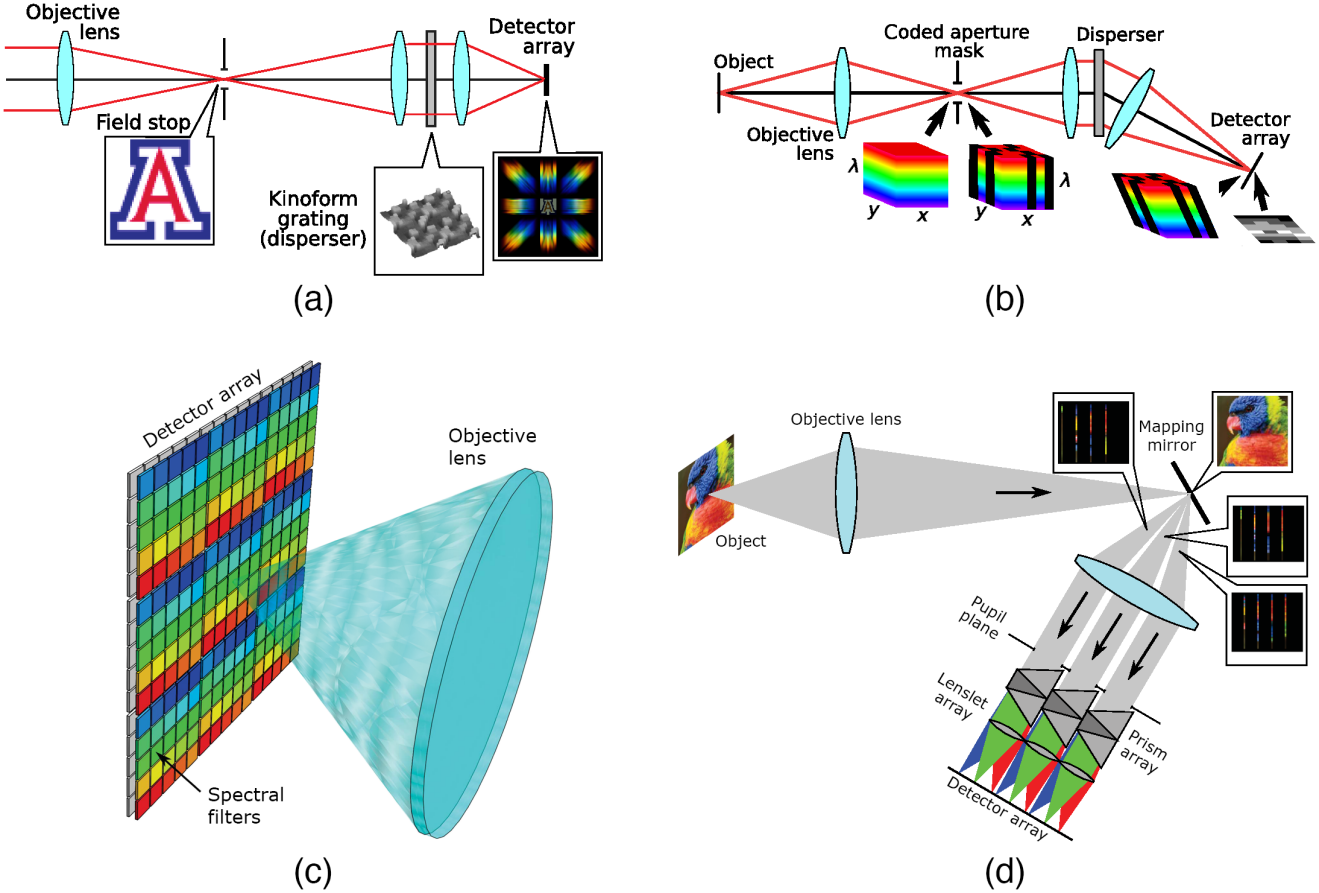
Optical layouts of some common snapshot imaging systems. (a) CTIS. (b) Coded-aperture imaging. (c) SRDA. (d) IMS (reproduced from Ref. 100).

The basis for CTIS was proposed in the early 1990s and was refined by Descour and Dereniak^11^ and Johnson et al.^104^ After passing through an objective lens, light enters the dispersive element of the CTIS, in order: an aperture, a collimator lens, and a grating/reflection dispersive device [Fig. 8(a)]. The aperture could be either a square or a slit depending on whether the desired purpose is imaging or line spectrometry. After passing through the dispersive device, a focusing lens focuses the light onto the staring sensor. What shows up on the sensor is a series of projections of the hypercube, arranged with the zeroth-order in the center and higher order further from the center. Dispersive devices were often transmissive devices, but reflective devices have been developed. Reconstruction from the projections slice is done using Fourier slice theorem. Reconstruction is more accurate with a higher number of projections. However, with limited sensor size, a higher number of projections also means that the reconstructed hypercube will have a lower spatial resolution. Due to advances in computing power, reconstruction-based systems such as CTIS were realized at lower costs. Habel et al.^103^ demonstrated a CTIS camera that uses DSLR cameras and low-cost components. Salazar-Vazquez and Mendez-Vazquez^102^ demonstrated an entirely open-source CTIS system with 3D-printed housing and off-the-shelf optical systems. Their imager had a significantly lower cost yet achieved a higher number of wavebands compared with previous CTIS cameras.

#### 5.3.2 Spectrally resolved detector array

While Bayer filters were common and can be manufactured for low-cost consumer electronics, the same cannot be said for SRDA used in snapshot cameras [Fig. 8(b)]. This comes down to the fact that Bayer filters used organic pigments or dye to color their filters, which are cheap but have large bandwidth.^106^ To manufacture SRDA with bandwidth narrow enough for accurate scientific uses, other types of interference filters must be used, including plasmonic filters, silicon nanowires, Fabry–Perot etalons, cavity-enhanced multispectral photodetectors, and multilayer quantum-well infrared photodetectors.^100^^,^^106^ Compared with architectures for spatial and spectral scanning cameras, SRDA were not typically robust for multiple applications. The number and value of wavelengths were fixed, which means that either the application must be specific or the SRDA must be custom-made, both limited the efficiency of research. Nevertheless, SRDA systems found many applications in bioimaging; we found systems that used SRDA in fluorescence microscopy,^23^ fluorescence endoscope imaging,^160^^,^^161^ skin imaging,^40^ and fundus imaging.^54^ Recovering the full spectral image from the acquired mosaiced image is not a simple task since the mosaic image shows a sparse representation of the captured data. Multiple authors have proposed generic algorithms to tackle this problem. Miao et al.^162^ proposed a binary tree algorithm to reconstruct the final spectral image. Wu et al.^163^ used sparse encoding to estimate reconstruction candidates, then used a heuristic method to search for the optimal reconstruction. Sawyer et al.^164^ compiled different established reconstruction algorithms and produced a Python package for open-source distribution.

There were two methods of manufacturing SRDA: one is by directly depositing and etching the filter layer on top of the sensor (monolithic integration), and another is by producing the filter layer separately and then mount it on top of the sensor (hybrid integration).^165^ On the monolithic integration end, a group of researchers in Belgium advanced many aspects of SRDA manufacturing using FPI.^76^^,^^79^^,^^117^^,^^121^^,^^166^ By depositing etalons of different cavity heights directly on top of the sensor, the resulting effect is like depositing interference filters of different bandpass values. Similar to the engineering of moving FPI, Bragg mirrors should be used in place of a single reflective material such as silver or aluminum.^117^ The materials varied; however, ${\mathrm{TiO}}_{2}$ and ${\mathrm{SiO}}_{2}$ were commonly used as high and low refractive materials.^167^ For a more in-depth article on the process of deposition and etch, refer Ref. 168. Out of this, the Belgian group developed many ultracompact commercial snapshot, spatial and spatial–spectral scanning cameras that use different interference filters patterns. For snapshot cameras, the filters were grouped into 4×4 square cells repeated throughout the entire sensors.^54^^,^^55^^,^^76^ For spatial and spatial–spectral scanning cameras, the filters changed bandpass values (and cavity heights) linearly across the sensor, resulting in a “staircase” pattern.^121^^,^^169^ Most recently, SRDA using etalons had been monolithically integrated on top of InGaAs sensors, making these types of spectral cameras also functional in the SWIR ranges.^170^^,^^171^ Elsewhere, other researchers were finding ways to bring the cost of manufacturing down. Yu et al.^172^ proposed a batch wafer manufacturing method that used silver and aluminum oxide (${\mathrm{Al}}_{2}O_{3}$) as the dielectric materials.

#### 5.3.3 Compressive sensing

Coded-aperture imaging cameras project the datacube onto the 2D imaging sensor through a coded mask [Fig. 8(c)]. In theory, it is not possible to resolve the images obtained from the coded aperture to the datacube since many different datacubes can produce the same image on the sensor. However, by applying some constraints onto the datacube such as sparsity and low variation, reconstructing a unique datacube became possible. Žídek et al.^173^ demonstrated that with off-the-shelf components and several custom optical lenses, a compact spectral camera using compressive sensing can be constructed.

#### 5.3.4 Image mapping spectrometer

The IMS (also called image slicing spectrometer) provides a one-to-one mapping of the datacube voxels onto the detector’s pixels. The captured image will be optically “sliced” by thin strips of mirrors. A dispersive element will then disperse the spectral elements of the image onto the sensor. The mirrors are arranged such that after dispersion, the spatial–spectral dispersion of the sliced image will fill the sensor [Fig. 8(d)]. IMS originated in astronomy and has been used in fluorescence microscopy^174^ and retinal imaging.^175^ In recent years, several compact systems that used IMS emerged. Bedard et al. built a compact IMS system that has high spectral–spatial resolution (350×355×41  pixels) and good acquisition rate (7.2  frame/s). Pawlowski et al.^176^ used lenslet array as the optical slicing component in their ultracompact snapshot system. The optical element measured only 27×27×8  mm, and the resolution was 210×279×54  pixels.

#### 5.3.5 Linear variable filter

An LVF is a monochromator that varies its dispersive property based on the spatial location. It is typically used in spatial–spectral imagers (see Sec. 3.3 for the “Snapscan” concept). Ding et al.^27^ adapted the concept onto snapshot imaging by incorporating lenslet (small lenses that project images onto a small part of the sensor). LVF is created using FPI of varying cavity height. The lenslet splits the image onto subimages, each of which falls onto a different part of the LVF. Snapshot imaging is achieved by combining spectral filtered subimages onto the datacube. Conceptually, this is similar to SRDA and suffers similar drawbacks as SRDA does.

### 5.4 Mechanical Components

Mechanical systems refer to nonoptical systems that are responsible for driving the optical components and provide the mechanical housing. Here, we discuss MEMS, which are commonly used in compact spectral cameras. MEMS refers to small systems that are manufactured using semiconductor fabrication techniques. These devices have been hypothesized as theoretically possible since the early 1960s^177^ and now encompass a large range of devices including RF switches, cantilever, piezoelectric, comb-drive actuator, and resonator.^178^ MEMS are electrically reliable, low on heat dissipation, and can be scaled up to large-scale and low-cost manufacturing processes.^8^ Prior to spectral imagers, MEMS has been used in portable spectrometers.^179^^–^^181^ The single most important process in the manufacturing of MEMS is photolithography, or the process of etching complex nanopatterns on a photosensitive polymer using lights.^182^ Photolithography enables MEMS to be produced in high volume and consistent quality. To integrate multiple MEMS components together, two main methods are used: surface micromachining (depositing the desired system on top of a sacrificial layer to be washed away) or bulk micromachining (directly shaping the substrate without the need for a sacrificial layer). In hyperspectral and multispectral imaging systems, both surface micromachining and bulk machining were used to produce MEMS.^157^ In FPIs, MEMS is used as the actuator that drives the distances between the two reflective surfaces. This can be accomplished using either piezo-actuators or electrostatic actuators.^183^ According to Trops et al.,^183^ piezo-actuators FPI had larger optical apertures and higher SNR, and electrostatic actuators were mass-producible, have smaller optical apertures, and lower SNR. In terms of spectral ranges, piezo-actuated MEMS FPI have a wider tuning range compared with electrostatic actuated MEMS FPI. Rissanen et al.^184^ and Näsilä et al.^159^ used surface micromachining to produce MEMS FPI spectral cameras that work with mobile phone cameras. Näsilä et al.^123^ used the same technique to produce a ultracompact (<40  g) spectral camera. Another type of MEMS is a digital micromirror device (DMD), which is an array of microscopic mirrors on a chip that can be individually activated and rotated. A common usage of DMD is in push-broom imaging, where DMD replaces the slit translation mechanism. The DMD selects the narrow section of the image and reflects that section onto the grating mechanism. This mechanism was seen in works by Arablouei et al.^142^ and Dong et al.^185^^,^^186^ A comb-drive MEMS was used by Wang et al.^187^ to rotate a mirror within their handheld spectral imaging camera. There are notable limitations to the range of movement in MEMS. On small scales, the mechanical strain and stress behavior is much different compared with their macrocounterparts. Pull-in phenomenon is seen when the electrostatic forces between MEMS elements are greater than the mechanical forces, which “pulls in” the MEMS components into each other and potentially causes a breakdown.^178^ In spectral imagers, this is most applicable to MEMS-enabled FPI imagers, where the air gap between the mirrors cannot be smaller than two-thirds of the initial unactuated air gap size to avoid pull-in phenomena.^159^^,^^183^^,^^188^

For compact devices that are planned to be used in outdoor situations, mechanical housing is as important as every other component. Crocombe^8^ discussed the importance of rigid and durable housing in portable spectrometers used in manufacturing. Even though devices in a clinical setting are not subject to harsh environmental factors the same ways devices in industrial or earth science fields do, mechanical housing is still an important factor in biomedical imaging devices. Many housing systems, if they are used in noncommercial devices, are likely to be highly customized using 3D printing. 3D printing has many advantages over traditional manufacturing, such as the ability to go from designs to prints within a short amount of time, the low cost of plastic, the lack of screws and adhesion, and the fact that when 3D printing different designs, the printer configuration remains largely similar.^85^^,^^189^ The rise in 3D printing of optical instruments is due to not just the large availability of 3D printers but also because of the open-source movement.^190^ For a review of 3D printing technologies and additive manufacturing, see Ngo et al.^191^ 3D printing can be used for many versatile components of spectral imaging. Ghassemi et al.^192^ and Cavalcanti et al.^59^ used 3D printing to develop biological phantom models for hyperspectral imaging. Ortega et al.^60^ created custom 3D printed gears to mechanically move the sample in a push broom microscopic imager. However, the most common use of 3D printing in spectral imager is for the housing of optical and dispersive elements.^85^^,^^102^^,^^131^^,^^159^^,^^187^^,^^193^ When researchers and hobbyists had access to 3D printers, design and manufacturing became an iterative process due to the speed and low-cost that 3D printing brings. Design was often done with the help of computer aided design (CAD) software. The most common 3D printing method used was fused deposition modeling (FSM), which uses a heated nozzle to fill semiliquid filaments in a layer-by-layer manner. Depending on the printer chosen, 3D printing using FSM can achieve high printing resolution and is capable of fitting optical lenses without much calibration necessary.^131^^,^^189^ However, FSM methods can have weak mechanical properties and poor aesthetic appearance compared with more advanced methods of additive manufacturing, such as stereolithography or power bed fusion.^191^ The most common material used was polylactic acid, commonly abbreviated as PLA.^191^ PLA is notable for having a low melting point and good biocompatibility. This aspect means that it is possible for researchers to use PLA to build biomedical components in their spectral cameras.^51^^,^^59^ However, PLA has some structural downsides. PLAs are known to shrink during printing; Sigernes et al.^85^ suggested that the actual design is 1% to 2% larger than intended so the optical components can fit. If the optical alignment is critical, 3D-printed housing can potentially produce optical misalignment and imaging artifacts. Despite shrinkage being a common problem in polymer-based printing, not many research studies discuss the solutions to counter this.^194^ Beyond accounting for the shrinkage in the initial design, Pearre et al.^195^ suggested bounding the printed components with solid structures. Alternatively, inkjet technology using plastic powder and cyanoacrylate adhesive was used by Wang et al.^187^ to print housing for their handheld spectral camera.

### 5.5 Electronics Components

Electronics is the driving component of all spectral imaging systems. “Electronics systems” refers to systems that provide illumination, capture images, control hardware, and transfer data. Systems that are expected to perform their work remotely, such as UAV-based, require a means to store power and to store data as well. Here, we discuss the use of illumination with a special focus on LEDs, the use of sensors, the use of microcontrollers, and the expected power consumption of the system.

#### 5.5.1 Power consumption

While it is still the norm for many laboratory-based imaging systems to have no battery systems and to instead draw power from the grid,^47^^,^^54^ many UAV-based systems require batteries to have reliable performance in a reasonable amount of time. Even though battery life is not typically specified in many commercial systems, a flight time of a UAV from 12 to 90 min^76^ gives a good idea of how long spectral cameras should operate. More well-known specifications that come with remote battery-powered spectral cameras are power consumptions, which range from 5 to 10 W,^134^^,^^137^ comparable to the power consumptions of UAVs (10 to 20 W) but still greater than that of many consumer products such as smartphones (<500  mW).

#### 5.5.2 Sensor technologies

Most digital imaging sensors used in compact spectral imaging fall into two broad categories: charge-couple devices (CCDs) and active pixel sensors, also called complementary metal oxide semiconductors (CMOS). CCDs use linked MOS capacitors to transfer electric charges from the pixels toward the shift register where it will be amplified. CMOS use MOSFET switches to access and amplify each pixel individually.^196^ The size and cost of CMOS benefit greatly from advances in semiconductor fabrication, which has been observed to double the amount of transistor every 2 years.^197^ However, CMOS have higher dark current compared with CCD.^198^ The majority of compact spectral CCD/CMOS use silicon as the semiconductor, which has an operational range between 550 and 900 nm. Shorter wavelengths with higher frequency in the SWIR (900 to 1700 nm) range can penetrate deeper into biological tissues and reveal more underlying features; however, capturing them requires alternative CCD materials, such as InGaAs.^25^^,^^158^ Alternative architectures for CCD and CMOS used in spectral imaging include intensified CCD^21^^,^^199^ and electron multiplying CCD.^200^

#### 5.5.3 Microcontrollers

Many spectral imaging systems use microcontrollers to provide system control. In recent years, open-source systems have made spectral imagers more low-cost and more customizable. These microcontrollers were part of the open-design movement, which aimed for free collaboration and sharing of schematic and software. Common open-source systems include microcontroller boards aimed at specific tasks (such as the Arduino Uno) and microcomputers capable of full-fledged control tasks (such as the Raspberry Pi). Programming these devices is much easier compared with previous generations of programmable circuit boards, as these newer devices used USB connections to transfer instructions. Furthermore, these devices have a large support community, making them the preferred option for off-the-shelf spectral cameras and for prototyping new designs.

Open-source microcontrollers in spectral imaging devices perform three main tasks: (1) drive optical components, (2) control illumination, and (3) act as a device to send and receive signals. Nevala and Baden^193^ used an Arduino Uno microcontroller in a spatial scanning camera to move mirrors on a predefined path. The microcontroller also drove the spectrometer via a transistor–transistor logic gate to capture data. Ortega et al.^60^ used a similar microcontroller to drive a stepper motor, which will translate the microscope stage for a line scan imager to acquire images. Näsilä et al.^159^ system used an MEMS actuator driven by an AC actuator signal. A microcontroller board used the $I^{2}C$ interface to drive the actuator. Some spectral imaging systems used narrowband LED to provide illumination, which the camera could capture the reflected light and construct the datacube in the same manner as a spectral imager does. However, to make acquisition fast, synchronization between LED and image capture was necessary. Ohsaki et al.^201^ constructed an LED flickerless system controlled by a Raspberry Pi microcomputer. Di Cecilia et al.^202^^,^^203^ used microcomputer to drive a pulse current to control the LEDs that was synchronized with the shutter: once the camera shutter was pressed, the microcomputer turned off the LEDs. Typically, the spectral imaging system was directly connected to the workstation through a USB connection, or the data were stored inside memory for later retrieval. Näsilä et al.^159^ prototyped an FPI-based spectral imaging system whose main controller is a Raspberry Pi microcomputer. The microcomputer sent signals to the FPI driver, received images from the camera module, and sent images through Wi-Fi to a workstation for further analysis. A similar setup was employed by Salazar-Vazquez and Mendez-Vazquez^102^ in their CTIS-based spectral camera. The microcomputer employed was also a Raspberry Pi, which sent commands to the camera module and sent images through Wi-Fi to the workstation.

### 5.6 Illumination

For most biomedical imaging applications, imaging was performed indoors using artificial illuminations. This posed various challenges for acquisitions. It is important to differentiate between luminance and radiance. Radiance refers to the quantity of radiant energy per emitted solid angle per receiving surface area and is applied across all wavelengths, whereas luminance is a human vision-centric measurement and is the radiance weighted by the response curve of the human eye. With spectral imaging devices, radiance is the more appropriate measurement of light sources whereas with regular cameras and human activities, luminance is more appropriate. Unlike outdoor illumination, which can exceed 100,000 lux in luminance, indoor systems using incandescent light only illuminate around 10,000 to 20,000 lux. Spectral imaging requires more illumination than similar RGB or monochrome cameras, because spectral cameras need to capture energy associated with a narrow range of wavelengths. Furthermore, many spectral imaging systems have additional filters that reduce the incoming light quantity. Some systems require wavelengths bandpass, lowpass, or high pass filters to block out unnecessary wavelengths.^54^ Some systems use beam splitters to either achieve snapshot imaging or to capture both live images and spectral images.^200^ Additional illuminations are often needed in spectral imaging acquisition. The additional components can increase the footprints of the system. The addition of light sources should be balanced with the applications. If the intensity of the light is too large, damage to the tissues can be irreversible. The most vulnerable organ is the eyes. Permissible exposure limit to the human retina is dependent on both the wavelength and exposure time. Rees and Dobre^204^ calculated the maximum exposure power to the eye at 0 deg to be ∼180  μW at 5 s and ∼120  μW at 30 s. For thermal light sources, Yan et al.^205^ evaluated maximum exposure for a direct angle to be $1.8\times 10^{-3}\times t^{0.25}\;J/{\mathrm{cm}}^{2}$ with time t in second and is $<3\times 10^{4}\;s$. These values are only for humans; for animals with retina diameter different than humans, the maximum permitted exposure needs to be varied. There is no definitive guidelines on animals’ retinal’ light exposure limits. For other organs, the limits are more forgiving and often orders of magnitude larger than many illuminations needs. Surgical lighting, for example, has a suggested luminance ranging from 40,000 to 160,000 lux,^206^ which is often enough for acquisitions in the visible light range.

There are different geometries to arrange the light source, the subject, and the imaging camera. Amigo et al.^207^ detailed several laboratory setups, which are frontal, lateral, contrast-transmission, diffuse, co-axial, and dark field. In each case, illumination should be as uniform as possible. Sawyer et al.^208^ detailed three types of nonuniformity: spatial uniformity, which refers to differences in illumination of incident light across object; angular uniformity, which refers to differences in incident and shadowed areas; and spectral uniformity, which refers to spatial uniformity across all wavelengths. They compared three different light setups for wide-field imaging: an LED ring, a fiber halogen ring, and a diffusion dome. They found that all systems achieved similar spatial and spectral uniformity, but the diffuse scattering dome achieved the highest angular uniformity.

For the illumination type, we covered the three commonly used types: halogen incandescent, gas discharged lamp, and LED. Halogen light operates in the ultraviolet, visible, and NIR regions. It offers a continuous spectrum suitable for acquisition with many bands. However, halogen light has a low color temperature (3200 to 5000 K) and can appear yellow at low illumination.^206^ Halogen and LED were the most often light sources in microscopes, so many spectral imaging applications that used microscopy also used halogen or LED as default illumination. Gas-discharged lamps, which include xenon lamps, mercury lamps, and mercury–argon lamps, often have higher color temperature. Xenon lamps have color temperatures ranging from 4000 to 6000 K, which makes them closer to outdoor lighting. Gas-discharged lamps often have energy spikes in the NIR regions, which can affect acquisitions. Due to these energy spikes, gas-discharged lamps are not used in retinal illumination and retinal surgeries. Both gas-discharged lamps and incandescent lamps emit a large amount of heat. If this is a concern, a fiber optic guide is needed to direct the light far away from the light source. Tunable laser is also seen in spectral imaging devices,^209^^–^^211^ although not in compact systems due to the size and power consumption of the laser components.

#### 5.6.1 Light-emitting diode

**Fig. 9:**
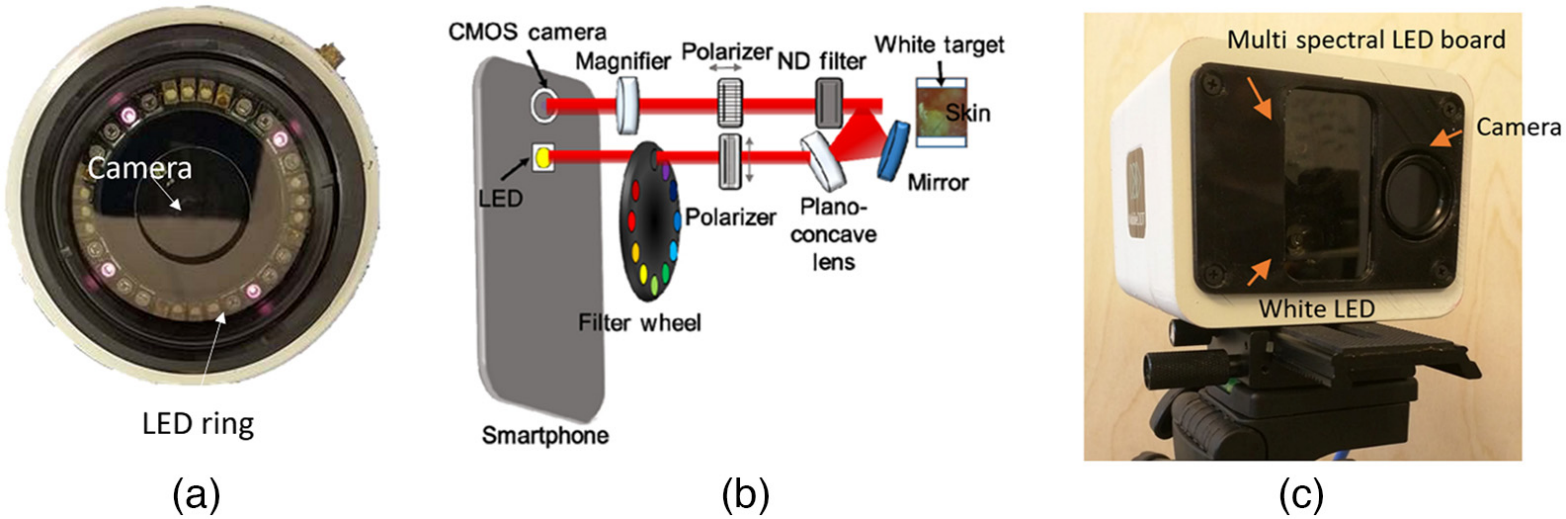
Different uses of LEDs in a compact imaging system. (a) A system that used 32 LEDs arranged in a circle. There were four of each types that are spaced 90 deg apart (reproduced from Ref. 24). (b) A system that used a singular white LED and a rotating spectral filter (reproduced from Ref. 22). (c) A system that arranged LEDs into a four-by-four rectangular plane (reproduced from Ref. 37).

The advantages of LED in illumination scanning systems are numerous: they are low-cost, fast, have low power consumption, low heat dissipation, and long life.^206^ A common way to use LED for spectral imaging is to use different LEDs to illuminate the object, each LED emits light within a specific waveband. Alignment of LED is important to achieve uniform illumination. Figure 9 shows different methods of arranging LEDs in a compact spectral imaging system. A common layout is the ring layout, where all LEDs are arranged in a circle.^22^^,^^24^^,^^25^^,^^38^ Li et al.^212^ proposed a different setup where the LEDs were arranged in a mosaic fashion, however warned that this arrangement can lead to nonuniform lighting for different wavelengths. Bolton et al.^37^ arranged the 16-LED setup into a 4×4 rectangular array. Light uniformity was ensured by choosing LEDs with very wide illumination angles. If the LEDs were arranged in a circle, it is better to have multiple of each type that are opposite to each other. Delpueyo et al.^24^ and Rey-Barroso et al.^25^ proposed a system using 32 LEDs, where four of each type of LED were arranged 90 deg apart [Fig. 9(a)]. For the acquisition camera, Shrestha and Hardeberg^213^ recommended that monochrome camera might perform better than commercial RGB cameras. The reason being that RGB cameras need to perform demosaicing operations, which altered the actual spectral readings. To control the LED, a separated power source and controller was needed to synchronize the LEDs with the acquisition camera. LEDs do not require a high-power source; a configuration built by Kim et al.^22^^,^^38^ only needed a 3.7 V battery to drive a multi-LEDs system [Fig. 9(b)]. If the LEDs emit different wavelengths, the activation voltage could be different for each LED.^37^ For Bolton et al.,^37^ different resistors values were required to produce different activation voltage using the same input voltage [Fig. 9(c)]. The biggest downside to LEDs is the limitations of wavelengths. The spectral imaging systems described can acquire only as many wavelengths as the number of different LEDs are available. For many researchers, this may not be a problem. Some applications only focus on specific excitation and absorption wavelengths and only need three to four different types of LEDs. Well-chosen LEDs are very beneficial. Another potential engineering problem comes from the quality of the LEDs. If the LEDs are bought from commercial vendors, then their bandwidth can range anywhere from 20 to 70 nm. If this is a problem, the interference filters should be applied to achieve a narrower bandwidth.^214^

## 6 Low-Cost and Compact Spectral Cameras

A large variety of compact cameras came not from commercial manufacturers but from academics and hobbyists who wanted to build custom imagers that are more accessible and more customized. Many low-cost systems needed to strike a balance between size, acquisition speed, spatial resolution, and spectral resolution. However, with recent advances in custom components, additive manufacturing, smartphones, and compact spectrometers, custom low-cost devices have improved in quality and compactness.

### 6.1 Commercial Off-the-Shelf Components

**Fig. 10:**
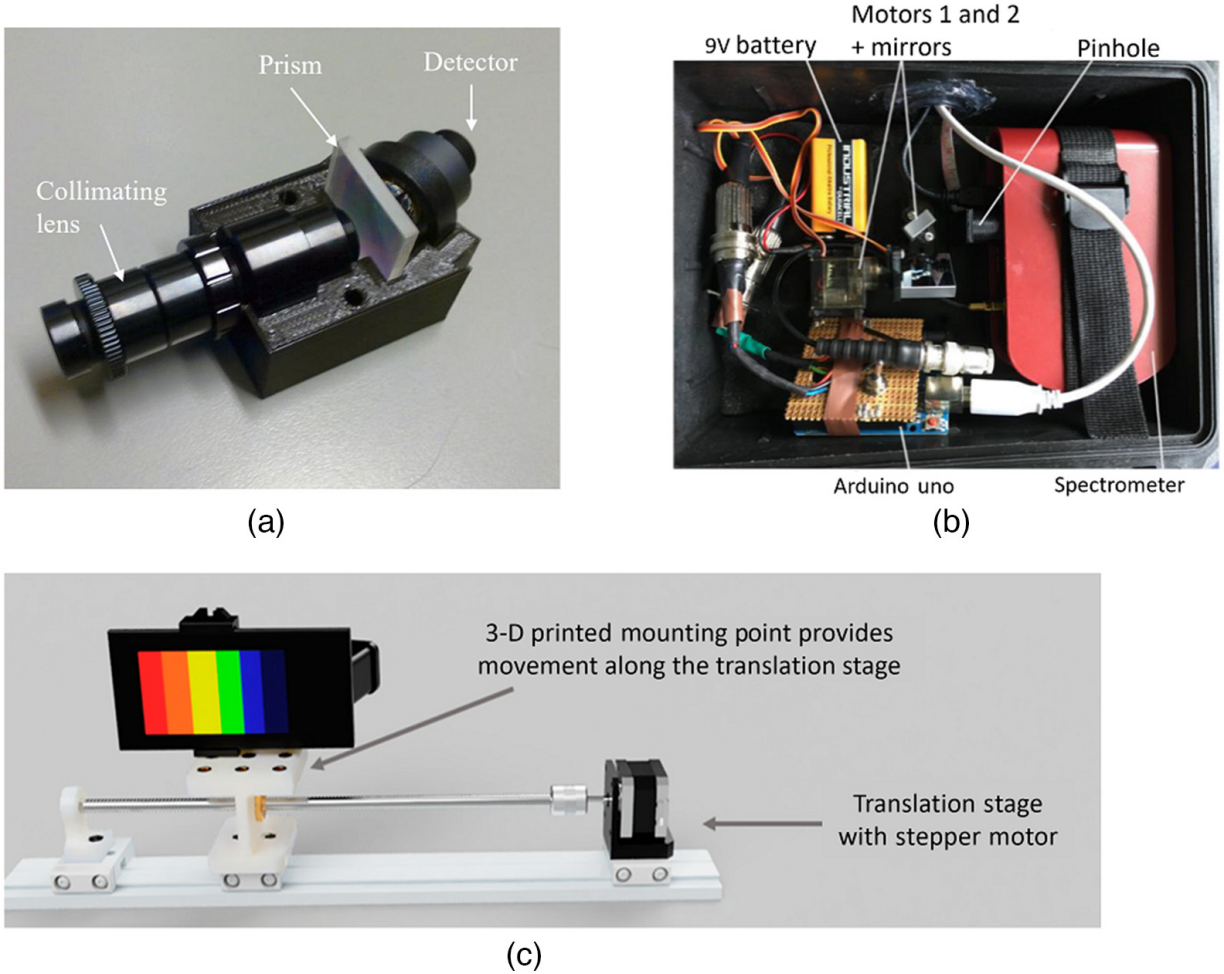
Spectral devices enabled using commercial off-the-shelf components. (a) A spatial scanning camera built using customized optical components and 3D printed housing (reproduced from Ref. 85). (b) A spatial scanning camera that uses portable spectrometers as the spectral acquisition device (reproduced from Ref. 193). (c) A smartphone-powered spectral imaging systems (reproduced from Ref. 215).

COTS, or commercial off-the-shelf, refers to components of a spectral camera that can be bought and assembled into a functional unit. Assembling COTS requires the researchers to have knowledge in building an optical system, electronics, control software, and hardware housing. The total price of all components added together is often much lower than the unit price of one commercial spectral camera of similar capability. Furthermore, COTS components allow researchers a high degree of customization. As mentioned in the previous section, components of a spectral imager include the optical system, the spectral dispersion system, the digital image detector, the control module/electronics, and the mechanical elements. It is more common for researchers to buy each of these components individually and assemble them together, because of the wide availability of low-cost options to choose from (Fig. 10). Alternatively, some researchers opted for portable spectrometers and digital cameras as the acquisition module and built the rest of their system surrounding those.

### 6.2 Optical Setup and Calibration

In the past, to conduct optical research, optical systems needed to be built on a rigid optical table and required large footprints. However, it is now possible to build optical systems with COTS components that are lightweight and have small spatial dimensions. Sigernes et al.^85^ [Fig. 10(a)] and Fortuna and Johansen^216^ built systems that were carried by UAVs. Their systems were spatial acquisition systems using transmission gratings. Kim et al.^22^ built a customized optical system to complement smartphone image acquisition. The system used a planoconcave lens, a reflective flat mirror, linear polarizers, and bandpass filters. The linear polarizers and the bandpass filters filtered the LED light sources of the smartphone, whereas the flat mirror and the planoconcave lens made the illumination broader and more uniform. Jeon et al.^217^ introduced a new type of diffraction optical element using photolithography and reactive-ion etching techniques. They used the fact that Fresnel diffraction is dependent on the wavelength to produce a diffractive element that produces spectral-varying point spread functions. 3D printed optical elements, such as reflective mirrors, focusing lenses, and prisms, despite already existing,^194^ have not been seen in any spectral imaging system as of yet. Customized mechanical housings are required to house these optical components. For that, 3D printing is the preferred option.

Even though COTS optical components are vastly cheaper than their commercial counterparts, lack of quality control can harm the acquisition quality. Smile, keystone, and chromatic aberration are all optical problems these customized systems face. We discussed smile and keystone in Sec. 5.1 in relation to spatial scanning systems. Chromatic aberration is a property of optical systems that makes the focus of different wavelengths not aligned. The point-spread function of the image depends on the wavelengths. Chromatic aberration makes focusing difficult. For spectral scanning systems, one method of dealing with chromatic aberration is to change the focus for each wavelength. However, this method could not be replicated with other types of spectral acquisition. A more robust engineering solution is to use achromatic lenses, which come at a higher cost than regular optical components. Mirror-based grating systems also reduce aberration; we discussed the Offner imaging spectrometer, which reduces aberration. If low-cost optical systems are to be expected to perform accurately, extensive calibration processes are necessary. Sigernes et al.^85^ discussed the steps to perform chromatic and sensitivity calibration in their customized camera. Riihiaho et al.^218^ performed smile correction by calculating the matrix that transforms curved spectral lines into the straight lines for each spectrum. Henriksen et al.^219^ proposed an algorithm that could perform real-time correction of smile and keystone based on matrix operations of calibrated results.

### 6.3 Portable Spectrometers

Compact and portable spectrometers have been developed and used for commercial, health, and scientific purposes.^8^ Many custom imagers in fact incorporated one or multiple commercially available spectrometers into their designs. By incorporating spectrometers, researchers did not need to worry about optical architecture and could focus more on the scanning methods. Spectrometers also have a much finer spectral resolution compared with line scanning spectral cameras. However, because the field of view of a spectrometer is typically small, the sampling scheme needs to be large with oversampling, otherwise the spatial resolution would be very low. Devices that used compact spectrometers were usually point-scanning or line scanning imagers. If only one spectrometer was used, the device would be like a point-scanning camera, and the researcher would devise an appropriate scanning path. Nevala and Baden^193^ equipped a commercial spectrometer that has effective spectral ranges from 350 to 950 nm with two mirrors controlled by Arduino microcontrollers [Fig. 10(b)]. Because the scanner had a circular window, they proposed circular scanning paths that are based on Fermat’s spirals. In the system proposed by Stuart et al.,^220^ light entered a focusing lens before being redirected by two movable mirrors into a spectrometer. Commercial spectrometers were used in confocal microscopy by Frank et al.^221^ who connected the confocal pinhole with a spectrograph using a fiber optic cable. If multiple spectrometers were used, then the device is a line scanning imager. Uto et al.^222^ used a series of eight spectrometers for a line scan imager.

### 6.4 Smartphones

Personal diagnostics with smartphones is gaining more attention in healthcare and bioengineering since smartphones are readily available, portable, offer high quality images, and are power efficient. This trend is reflected in spectroscopy and spectral imaging alike. Crocombe^8^ outlined four main usages of smartphones in spectrometry and spectral imaging: (1) to receive data from the spectrometer as a separated module; (2) to receive data; (3) to send command to the optical module; and (4) to be the optical module itself. The fourth usage is only applicable in the visible and near-infrared regions, as silicon detectors used in CMOS and CCD only operate within the 400 to 1000 nm wavelengths. Spectroscopic solutions have been developed for the smartphone for the purposes of food quality control,^223^ lab-on-a-chip diagnosis,^224^^,^^225^ and fluorescence spectroscopy.^226^ Similarly, spectral imagers have been developed with smartphones in mind for biomedical, remote sensing, and field laboratory purposes. Kim et al.^38^ developed a system that used smartphones as the controlling center for their MSI. In their system, the smartphone sent signals to a microcontroller unit that drove LEDs and received photos from a CMOS camera. Näsilä et al.^159^ and Rissanen et al.^184^ developed FPI-based spectral imagers with smartphone as the centerpiece, both as a controlling device that sent and received signals and as the optical camera itself. Smartphone cameras are extremely powerful and fast, which makes them the ideal candidate for spectral imaging modules. If the devices are used in personal healthcare settings, custom parts need to be developed for smartphone spectral imaging to fit the specific needs. Stuart et al.^215^ built a spectrograph with the smartphone camera as the acquisition device [Fig. 10(c)]. Bolton et al.^51^ and Cavalcanti et al.^59^ used 3D printing to create custom smartphone-based spectral colposcope and otoscope, respectively. Their systems used LEDs driven by microcontrollers to provide narrowband illuminations. These designs also used the smartphone’s ability to transfer signals over Bluetooth or Wi-Fi to construct an internet of things (IoT) system for spectral images acquisition and analysis. In some applications, smartphone can also be used as a geo-tagger, providing GPS data along with images to register images together.^227^

### 6.5 Open-Source Design

Open-source design refers to the creation of software and hardware with the intention of freely sharing those designs for others to use, study, modify, and even commercialize without concerns of copyright or patent infringement. This allowance is performed through many different forms of open-source licenses. Whereas the nature of software makes open-source software easily distributed, open-source hardware still requires usages of tangible components to manufacture and assemble. Ideally, open-source hardware designers would want to use low-cost components, 3D-printed components, or other open-source hardware components and run free open-source software. Some spectral imaging researchers published systems that were open source with the goal of easily sharing research progress. As an example of the open-source model, Riihiaho et al.^218^ built a 3D-printed spectral imaging camera with designs from Sigernes et al.^85^ Some further modifications were made to reduce smile and aberration, which were publicly shared along with 3D CAD files. They also built software that performed aberration correction and shared it publicly. Nevala and Baden^193^ published the entire hardware and software design of their system, along with raw spectral data captured by the camera. Low-cost spectral imaging was driven not just by open-source hardware but also through open-source software. Many researchers published hyperspectral processing software as free and open source. Berisha et al.^228^ published a framework for processing large-scale biomedical hyperspectral images at a faster speed using GPU. The entire software and all its algorithms were published under a license that allowed inclusion in both free and commercialized systems.

## 7 Applications of Compact Spectral Cameras in Biomedical Imaging

Many bioimaging researchers use compact and ultracompact cameras in both laboratory settings and clinical settings. Systems have been built for light-field microscopes,^32^^,^^43^^,^^47^^,^^60^ confocal imaging microscope,^229^^,^^230^ fundus cameras,^54^^,^^55^ endoscopes,^46^^,^^48^^,^^56^^,^^140^^,^^200^^,^^231^ laparoscope,^63^^,^^65^^,^^66^^,^^70^ colposcope,^51^^,^^52^ and otoscope.^59^ Spectral imaging for biomedical purposes has its own specifications that influence the choice of systems. Most commonly, images are captured in the visible-near infrared range (VIS-NIR) from 400 to 1500 nm. Imaging in the visible range often leverages the contrast between different types of dyes and stains, whereas imaging in the infrared range often leverages pure biochemical signatures of biotissues.^232^ VIS and NIR camera sensors need to be separated, with the first being silicon-based and the second being InGaAs-based. The spatial resolution should be enough to differentiate key features. In head and neck cancer (HNC) diagnosis, for example, the positive margin (margin of cancer plus normal tissue) for cancer identification is around 5 to 10 mm.^233^ The spatial resolution can be measured with an optical target, such as the USAF target. If the spatial resolution of the camera is not enough, magnification devices can be used. Acquisition speed can be slow for *ex vivo* applications but must be fast for *in vivo* applications. In a spectral colonoscopy system developed by Kumashiro et al.,^48^ they found that the acquisition time cannot exceed 5 s, otherwise the image quality will be unusable. Fast acquisition time also means that the illumination must be higher to accommodate it. In surgical or clinical settings, systems must be sterilized or isolated from the environment. Drapes, resin, and sealants can be used in these situations.

### 7.1 Diagnosis and Monitoring of Diseases

The primary diagnostic targets for spectral imaging researchers were the skin, the oral cavity, and the retina. These organs are the most accessible organs for *in vivo* diagnosis. Using endoscopic systems, the luminal organs of the lower abdominal regions could also be imaged with spectral imagers. However, research in this field *in vivo* was scarce because *in vivo* research of lower abdominal regions requires systems with fast acquisition time and low illumination. For *ex vivo* applications, most spectral imaging research was done on pathology slides or tissues. *Ex vivo* tissues were imaged either in a tabletop setting using reflectance data or through a microscope, which can be reflectance or transmission data.

#### 7.1.1 Skin cancer

**Fig. 11:**
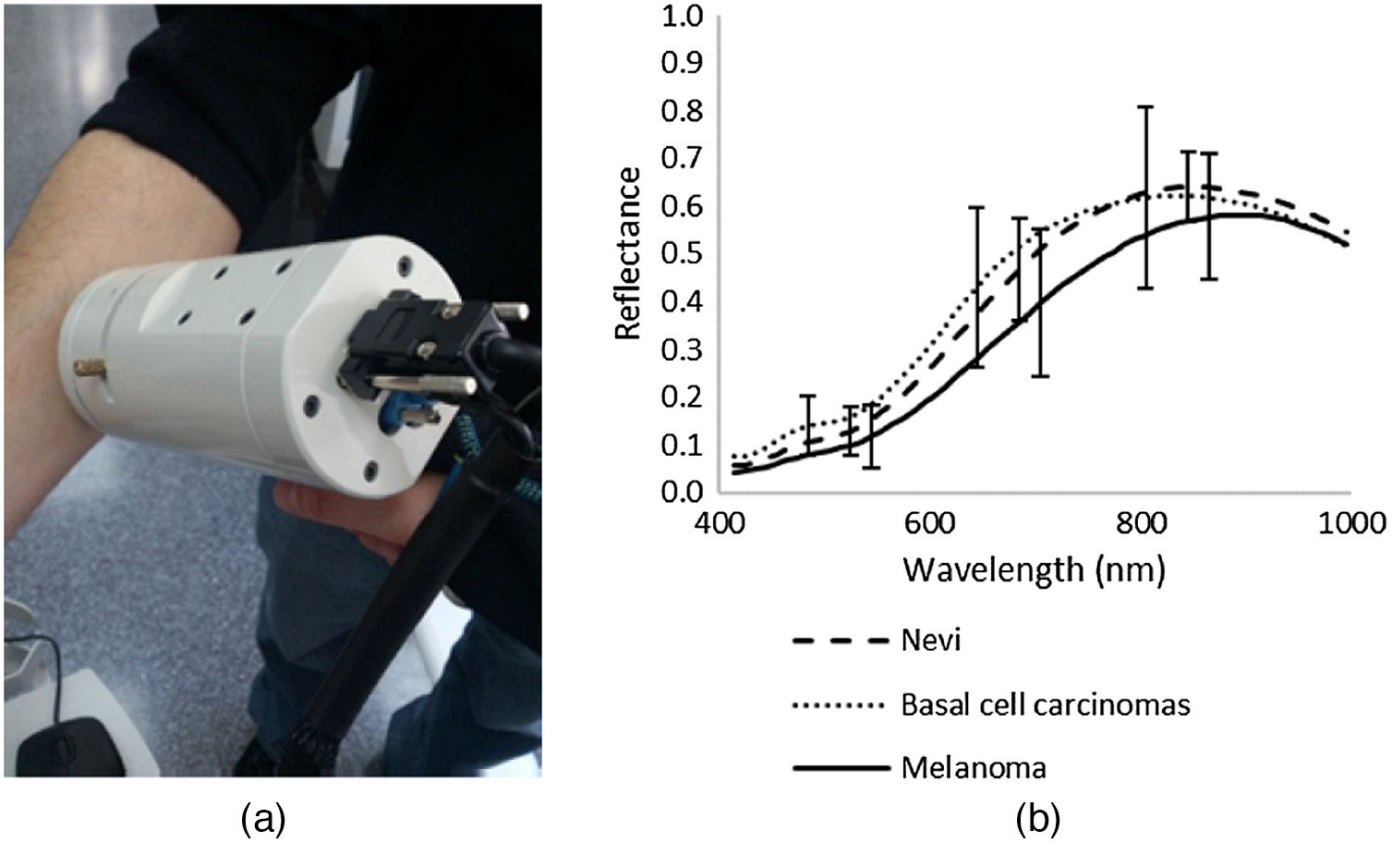
(a) A compact and handheld spectral imaging system that used LEDs to provide spectral scanning. The system weighed 500 g and could capture images in eight wavelengths. (b) Using the system described in (a), different spectral signatures could be seen for nevi, melanoma, and basal cell carcinoma (reproduced from Ref. 24).

Both melanoma and nonmelanoma skin cancer (MNSC), which include Bowen’s disease, Kaposi’s sarcoma, basal cell carcinoma (BCC), and squamous cell carcinoma, are on the rise.^234^ While MNSC is more common, melanoma has a higher mortality rate.^235^ Early diagnosis and removal of both melanoma and MNSC are necessary. However, many forms of skin cancer appear similar to benign neoplasm.^236^ Dermatoscopy can be used to differentiate benign from malignant skin lesions; however, dermatoscopy requires clinician input. Spectral imaging has been used to improve automatic classification with high sensitivity.^236^ In early 2000, Hewett et al.^21^^,^^237^ developed a portable imaging station to determine tumor margin of MNSC lesions. They used 5-aminolevulinic acid (5-ALA) to induce the fluorescing molecule protoporphyrin IX (ppIX). After application of 5-ALA to the skin, fluorescence imaging of MNSC lesions was taken at the 400, 540, 600, and 635 nm wavelengths. Their results showed a clear outline of the lesion at the 635 nm wavelength, which corresponds with maximum ppIX fluorescence. In the same time period, Elbaum et al.^236^ developed a handheld spectral imaging system to automatically classify melanoma and melanocytic nevi (pigmented nevi, moles). From a set of 10 spectral images obtained from 430 to 950 nm wavelengths, 822 candidate parameters were extracted in wavelet domain and spatial domain. The features were supposed to represent morphological and textural elements of the lesions. Through training an expert system using a dataset of 63 melanoma images and 183 melanocytic nevi images, they achieved 100% sensitivity at 85% specificity. A similar handheld system [Fig. 11(a)] was developed by Delpueyo et al.^24^ to classify melanoma and BCC from pigmented nevi [Fig. 11(b)]. First-order statistics were used to extract morphological features. Their systems achieved 91.3% sensitivity at 54.5% specificity for both melanoma and BCC. Rey-Barroso et al.^25^ continued the study with a second handheld camera that can image in the NIR region (995 to 1613 nm). Higher wavelengths penetrated deeper through the skin and showed more features pertinent to skin cancer diagnosis. With the same set of features, they produced a sensitivity of 85.7% at specificity 76.9%, which was an improvement in specificity over the results obtained by Delpueyo et al. without much loss in sensitivity.

Many melanoma diagnostic tools that use spectral imaging exist on the market. However, they have high cost.^238^ Several researchers developed smartphone solutions for identification of malignant melanoma that were low-cost and accessible. Kim et al.^22^ built a smartphone spectral imaging system that photographed in 10 wavebands from 440 to 690 nm. They also created a software platform that identified lesions margin and graded lesions severity from spectral images. Ding et al.^27^ built a similar snapshot system that used smartphone camera. They photographed nevus lesions and identified elevated optical density of the nevus region in the 550 to 640 nm wavelengths; these wavelengths correspond to the peak absorption wavelengths of melanin and oxygenated hemoglobin. Uthoff et al.^28^ developed a smartphone spectral imaging system that mapped oxygen concentration, melanin concentration, and erythema measurement onto images of squamous cell carcinoma. However, these methods were only able to visualize the outline of skin lesions. To show that these smartphone-based methods can classify between malignant and benign melanoma lesions, further clinical studies are necessary.

#### 7.1.2 Head and neck cancers

**Fig. 12:**
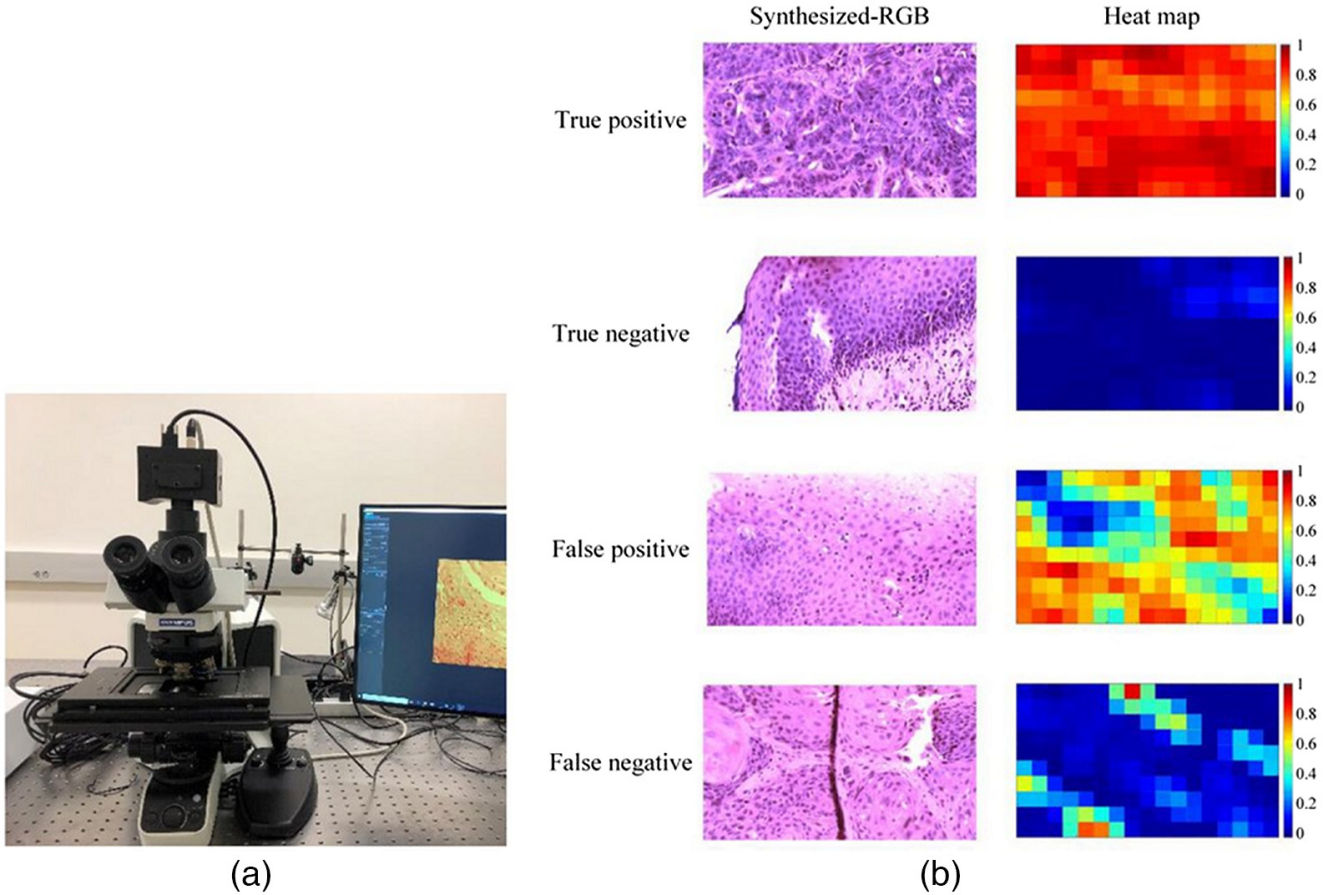
(a) A compact hyperspectral Snapscan camera on top of a microscopic system (reproduced from Ref. 47). (b) Using the system described, a series of hyperspectral digital pathology images were acquired. The slides were from patients diagnosed with squamous head and neck carcinoma. A machine learning system was used to produce a probability heat map of cancer occurrence (reproduced from Ref. 240).

HNC refers to cancers that originate from the nasal and oral cavity, nasopharynx, oropharynx, hypopharynx, larynx, and esophagus. Up to 90% of cases of HNC are squamous cell carcinoma,^239^ so the majority of studies reviewed focus on head and neck squamous cell carcinoma (HNSCC). Liu et al.^45^ used an AOTF camera to perform tongue tumor pixel segmentation. For classification, they used the sparse representation method. Their system achieved 96.5% pixel-accuracy on a dataset of 65 tumor images. Bedard et al.^46^ built a snapshot imaging system for imaging both autofluorescence and reflectance data. They used the system to photograph images of the oral cavity in healthy individuals and in patients with oral cancer. Using spectral unmixing, they were able to (1) highlight vasculatures in the lip region and (2) determine boundaries of tumors in the oral cavity. To image further into the oral cavity, spectral imaging systems used endoscopes. Often, in *in vivo* diagnosis, perfusion is a feature of interest since higher perfusion could be an indication of neoplasm. Köhler et al.^70^ created a spectral laparoscopic system for imaging the esophagus. Using customized metrics, they were able to visualize the hemoglobin index of a resected esophagus from a patient with Barrett’s syndrome. Our research lab contributed new research in identification of HNSCC *ex vivo* with the use of a compact spectral camera (Fig. 12). Ma et al.^47^ imaged histologic slides resected from the laryngeal and hypopharyngeal regions of patients with HNSCC [Fig. 12(a)]. They proposed two different methods of classification: (1) a support-vector machine (SVM) method that uses spectra of segmented nuclei as input data and (2) a convolutional neural network method that uses small image patches as input data [Fig. 12(b)]. They found that the CNN classifier had better performance compared with the SVM classifier. However, they also found that classifiers trained on spectral images did not outperform classifiers trained on RGB images.

#### 7.1.3 Lower abdominal cancer

Diagnosis of cancerous tumor in the lower abdominal region incorporates endoscopic tools. Kumashiro et al.^48^ used a commercial compact spectral camera for both *in vivo* and *ex vivo* identification of colorectal cancer tumors. For *ex vivo* observation, the camera was connected to a stereoscopic microscope. For *in vivo* observation, the camera was connected to a colonoscope. They found that in both *ex vivo* and *in vivo* analyses, the absorption rate of healthy mucosa tissues at 525 nm wavelength was significantly lower than that of adenocarcinoma. For *in vivo* data, tumor classification and real-time tumor mapping were also attempted. A sensitivity of 75.0% was achieved. Erfanzadeh et al.^50^ and Zeng et al.^49^ developed a handheld spectral imaging system for classification purposes. Erfanzadeh et al. imaged resected ovaries and found that the system can potentially identify malignant tumors from benign ovarian cysts. Zeng et al. used the system to image resected colorectal tumors. Mink et al.^52^ recognized the need for low-cost tools for cervical cancer diagnosis and developed a smartphone-based spectral colposcope. The system showed promise in augmenting biopsy and regular colposcopy. Baltussen et al.^66^ used two different spectral cameras, one operating in the visible light range (400 to 1000 nm) and one operating in the NIR range (900 to 1700 nm). Four types of tissues were classified: fat, healthy colorectal wall, colorectal tumor, and mucosa. They found that the near-infrared camera slightly outperformed the visible light camera in terms of classification result. Sun et al.^241^ produced a dataset of 880 hyperspectral images of cholangiocarcinoma (cancer of the bile duct) using an AOTF-based system. They used two types of CNN (Inception-V3 and ResNet-50) to classify cancerous tissues from normal tissues and achieved a 2% increase using hyperspectral data over using RGB data.

#### 7.1.4 Other types of cancers

In many forms of solid-tumor cancers, surgery is often the necessary treatment. It is important that surgical resection remove all malignant tissues. Spectral imaging on *ex vivo* tissue could be a method to identify or improve cancer/normal margin. Van Manen et al.^53^ used a snapshot camera to image excised breast tumors. They found that on average, tumor tissues had significantly higher fluorescence intensity compared with healthy tissues from 450 to 950 nm wavelengths, except at 619, 629, 897, and 934 nm wavelengths. Using the hierarchical stochastic neighbor embedding algorithm, they reduced the number of wavelengths as features and improved segmentation accuracy. Ortega et al.^242^ combined a compact spatial scanning imager with an upright microscope and custom scanning stage. They used their custom system to generate a database of hyperspectral brain tumor histology. For classification between tumor and normal regions, three different supervised classification methods were used: linear neural network, SVM, and random forest classifier. They found that the neural network classifier has the best overall accuracy of 78.2% while achieving a sensitivity of 75.44% at 77.03% specificity.

#### 7.1.5 Healing from burns, scars, and wounds

External factors such as age, lifestyle, smoking, and diet influence the healing process. Homeostatic imbalances such as ischemia (low perfusion), hypoxia, edema, and necrotic tissues also directly affect healing. To gauge healing process from burns, scars, and wounds, constant monitoring of perfusion, oxygenation rates, and tissue formation must be performed along with visual analysis.^243^ Methods used for wound monitoring included angiography with indocyanine green dye,^244^^–^^246^ LSCI,^247^^–^^249^ optical coherence tomography,^250^^,^^251^ laser Doppler flowmetry,^252^ and high-resolution ultrasound imaging.^253^^,^^254^ While no method offers distinct advantages over others, reflectance spectral imaging offers compact hardware and low-cost, noninvasive imaging.

**Fig. 13:**
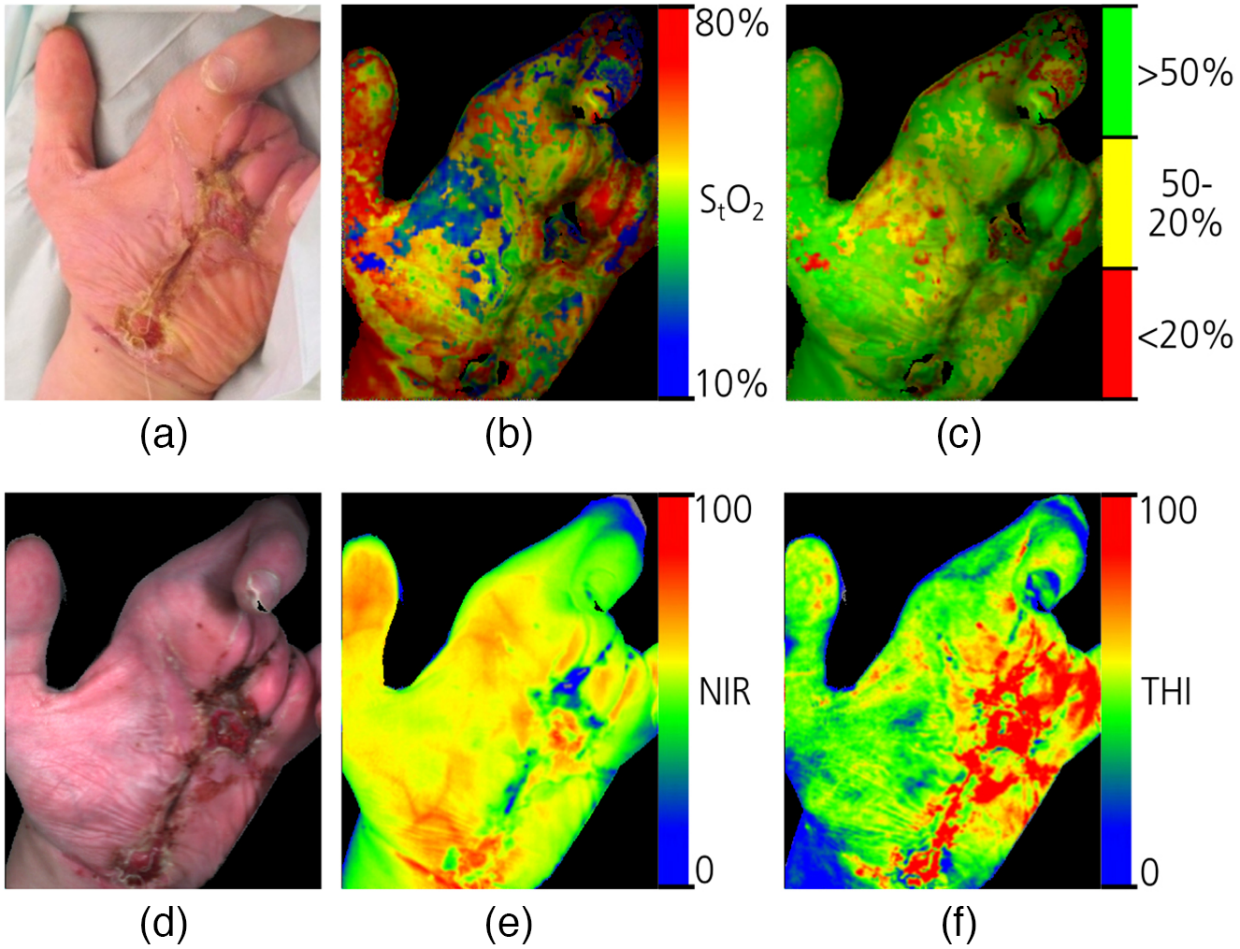
Estimation of physiological values from a wound photograph. (a) RGB image, (b) and (c) relative and segmented tissue oxygenation mapping ($S_{t}O_{2}$ in the figure), (d) reconstructed RGB image from hyperspectral data, (e) near-infrared-based perfusion data (NIR in the figure), and (f) relative tissue hemoglobin index (THI in the figure) (reproduced from Ref. 257).

Marotz et al.^255^ and Holmer et al.^256^ used a compact push-broom camera that operated in the VIS-NIR region for the purpose of assessing skin transplantation wounds. Reflectance data in the visible region were used to calculate hemoglobin relative concentration and oxygenation rate in the superficial dermis layer [Figs. 13(b) and 13(c)]. On the other hand, near-infrared reflectance data revealed deeper circulation in the subcutaneous layers and were used for estimating deep perfusion [Fig. 13(e)]. These data were mapped over the wound to assess the presence of ischemia [Fig. 13(f)]. Kulcke et al.^34^ later used the same commercial spectral system to image wound healing over a period of 2 weeks and showed that the perfusion and tissue oxygenation rates reduced to normal level after 2 weeks. Rutkowski et al.^33^ used compact spectral camera to monitor wound healing treatment with cold atmospheric plasma. They showed improved angiogenesis in both the dermis and subcutaneous tissue layers through cold atmospheric plasma treatment *in vivo*.

#### 7.1.6 Pressure sore and vascular occlusion

Pressure soreness, also called pressure ulcer or bedsores, is a pathological disease where the skin is injured from continuous pressure. Pressure sore is common among bed-ridden patients and often develop around areas with bony protrusions such as the heels, tailbone, hips, and ankles.^258^ To prevent pressure sores, monitoring of vascular occlusion is important.^259^ Van Manen et al.^39^ combined snapshot spectral imaging with LSCI to image the upper arm during and after occlusion. During occlusion, perfusion in the epidermis decreased, which was detected with LSCI. From spectral imaging, oxygenation rate was determined. They found that oxygenation rate measured with spectral imaging correlates with perfusion measured with LSCI. Their findings showed that spectral imaging can be helpful in monitoring epidermal blood flow. Klaessens et al.^260^ developed a compact spectral scanning system using LCTF to image constricted hand in the 420 to 730 nm wavelengths. During occlusion, they showed that the concentration of deoxygenated hemoglobin and oxygenated hemoglobin increased and decreased, respectively. After the occlusion was released, the absorption and oxygenation level overshot before stabilizing to regular level after a short period of time. He and Wang^42^ used smartphones to record hyperspectral imaging of fingers under pressure and showed similar results. Chang et al.^31^ used a compact commercial snapshot camera to measure the oxygen saturation and monitor bed sores in enrolled patients. A condition similar to pressure ulcer is foot ulcer, which is the ulceration that happens below the ankle. Foot ulcer is common among people with diabetes and can be chronic in nature.^261^ Foot ulcer can also be monitored by monitoring oxy- and deoxysaturation for both healing and prevention.^262^^–^^264^ Yang et al.^265^ developed a compact push broom imager that analyses oxygen saturation to predict the healing quality of foot ulcers. Yudovsky et al.^266^ developed a custom spectral imaging system using LED that illuminated at 15 different wavebands between 450 and 700 nm to analyze oxy- and deoxysaturation. The researchers used the system to predict foot ulcers before they can form. Lee et al.^267^ performed a pilot study using two commercial spectral cameras, one in the hemoglobin absorption wavelengths (542 to 578 nm) and one in the near-infrared spectrum (760 to 830 nm).

#### 7.1.7 Retinal diseases

Fundus imaging poses special engineering challenges for compact imagers. Because the eye is a small organ with limited reflection spectra, fundus imaging requires both illumination and magnification for accurate assessment.^268^ Many different biomarkers and diseases can be detected from the spectral response of the eyes. In clinical settings, most fundus cameras are table-top bulky systems, but many compact handheld and smartphone-based systems have been developed; for reports on these compact systems, consult reviews by Panwar et al.^269^ and Wintergerst et al.^270^ In humans, the macula is covered by the macular pigments lutein, zeaxanthin, and meso-zeaxanthin. These pigments are known to contribute to visual acuity.^271^ The appearance of this pigmented region can serve as biomarkers for visual or neurological diseases and is often quantified by macular pigment optical density (MPOD). Diabetic retinopathy is a complication of diabetes that affects the vessels of the retina and can cause blindness in some cases. Oxygen saturation of eye vessels can be used to infer progression of retinopathy *in vivo*.^272^ Early explorations in using compact spectral cameras for oxygenation monitoring was done by Johnson et al.^104^ through a custom-made CTIS. The system was designed to capture 50 spectral bands within the 450 to 700 nm range. Using the snapshot camera, they were able to demonstrate age-related changes in retinal vessels’ oxygen saturation in two healthy volunteers 30 years apart in age. A CTIS system was later used by Fawzi et al.^58^ to estimate MPOD in place of other methods, such as autofluorescence imaging and Raman spectroscopy. Li et al.^55^ used a compact SRDA camera with a microscope to image rat retina. The authors demonstrated that oxygenation rate can be successfully extracted from the snapshot camera. Kaluzny et al.^54^ continued the study in human subjects. By connecting the camera with a table-top fundus imaging system, they were able to image human retina in spectral datacubes. Using a best-fit model, they estimated oxygen saturation rate in the retinal arteries and veins. Through repeated imaging of the same eye, they achieved a mean standard deviation of 1.4%, showcasing high repeatability of the system. They also estimated MPOD, demonstrating that the snapshot system can extract multiple physiological data from only one measurement.

#### 7.1.8 Diseases of the central nervous system

**Fig. 14:**
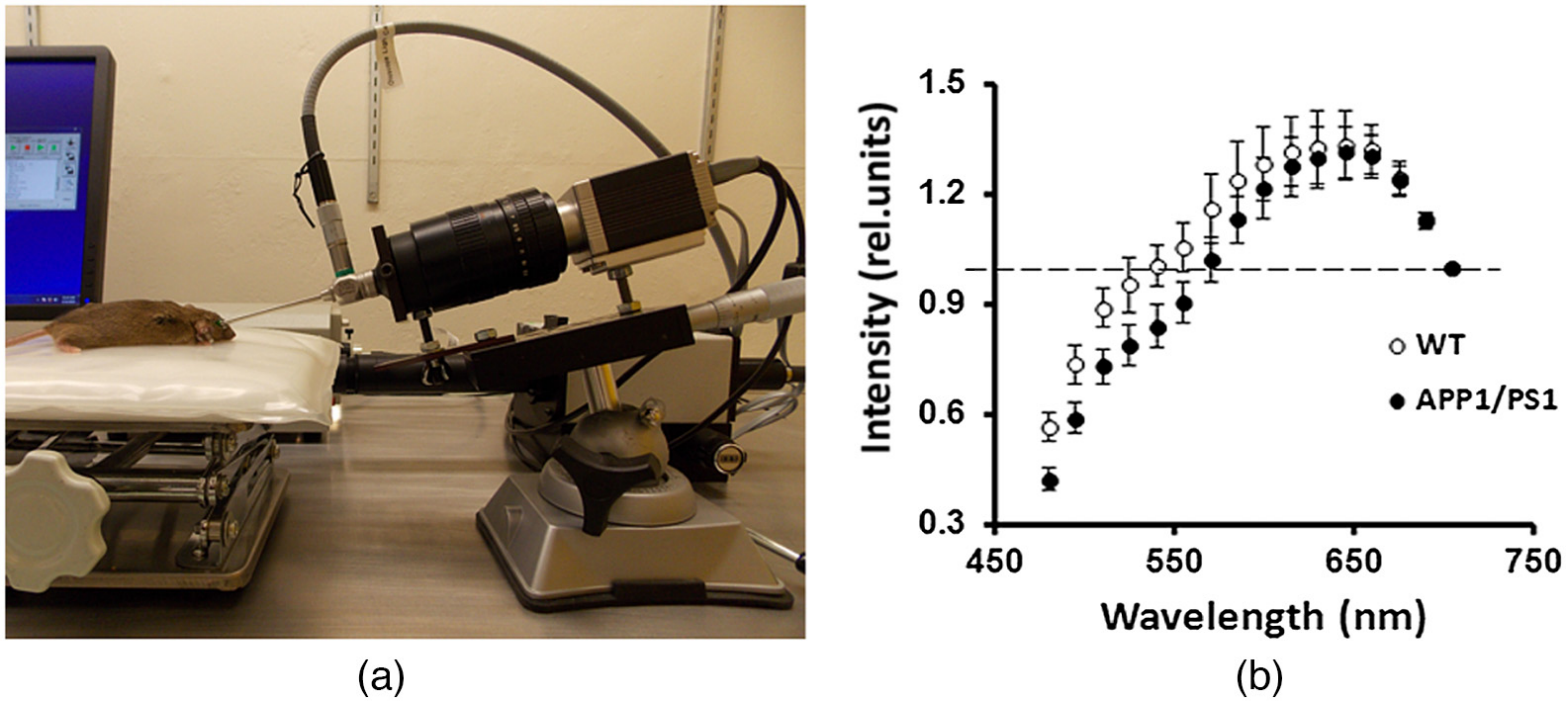
(a) A compact spectral imaging system used to image the retina of mice. (b) Using the system described, the retina of wild-type mice (WT) and transgenic mice for Alzheimer’s disease (APP1/PS1) were imaged. The result showed a significant difference in the spectral signature between the two (reproduced from Ref. 56).

An exciting recent application of spectral imaging in bioengineering is the monitoring and diagnosis of neurological diseases through retinal imaging. It is known that the retina contains optical neurons that are directly linked to the brain, so it follows that biomarkers for many neurological diseases, such as Alzheimer’s disease (AD), Parkinson’s, and multiple sclerosis can be seen through retinal imaging.^273^ Amyloid beta (Ab) peptide has been identified in the brains of people with AD and is a known biomarker. Similarly, autopsy of patients with AD shows high concentration of Ab in the retina.^274^ Through spectral imaging of transgenic Alzheimer’s mice using a compact endoscope, More et al.^56^ showed that in the wavelengths from 450 to 700 nm, there existed a marked difference in the optical spectra between wild-type mice and transgenic mice (Fig. 14). They also showed that changes in optical spectra strongly correlated with the accumulation of Ab in the retina and progression of AD over time.

### 7.2 Surgical Guidance

Lu et al.^7^ identified the four key benefits of using spectral imaging in surgical guidance: (1) visualization of microsurgery features, (2) hyperspectral tumor segmentation during resection, (3) monitoring of tissue oxygenation rate, and (4) visualization of large organs. The use of compact spectral imager benefits surgical guidance greatly because they free up limited space in the surgical room. Because spectral imaging is good at identifying blood oxygenation status, many researchers used them in the surgical room to monitor blood flow. Anastomotic insufficiency is a break or leak in a surgical suture and is among the most serious complications in colorectal surgery. Jansen-Wilken et al.^69^ used a spectral camera to detect the anastomosis site during small bowel surgery. Many other researchers explored similar surgical complications, most commonly ischemia.^63^^,^^67^^,^^275^ Akbari et al.^67^ used two cameras that operated in the 400 to 1000 nm and 900 to 1700 nm wavelength for bowel surgery. They found that the highest contrast between normal and ischemic regions in the intestine were seen in the 765 to 830 nm wavelength range, and they used SVM to evaluate ischemia progression over time.

#### 7.2.1 Spectral endoscopy

**Fig. 15:**
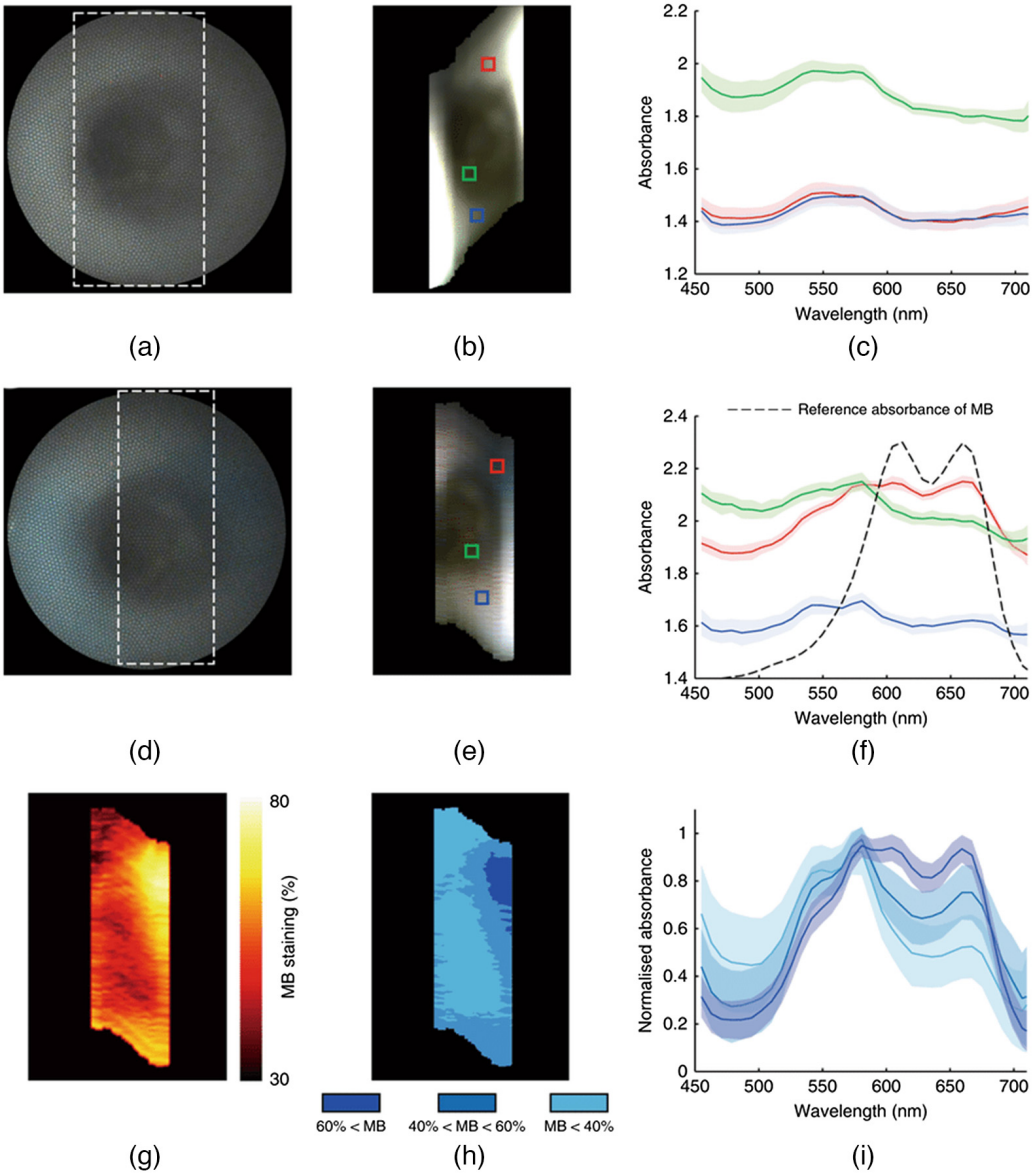
Images taken from an *ex vivo* pig esophagus by a compact spectral endoscope. (a)–(c) The esophagus taken before staining. (d)–(f) The esophagus after MB staining. (g) and (h) The estimation map of MB concentration using two different linear unmixing methods. (i) The spectral absorbance plot for the shaded areas in (h). In (c) and (f), the color corresponds to the color segment in the corresponding image to the left (reproduced from Ref. 231).

Many researchers investigated the use of spectral imaging in combination of surgical visualization tools. Endoscopic tools, which aid minimally invasive surgery, are often combined with compact spectral cameras. Kumashiro et al.^48^ attached a mobile spectral camera to a colonoscope. They directly observed colon lesions *in vivo* during biopsy. However, because the camera had a long acquisition time, scanning time was limited to 5 s and scanning resolution was limited to 200×200  pixels. Nevertheless, they found significant differences in absorption between normal mucosa and adenocarcinoma regions at the wavelengths 525 nm. Laparoscopy is an operation performed through small incisions with the aid of cameras and is minimally invasive. Clancy et al.^63^ built a custom laparoscope and measured bowel oxygenation rate during clamping *in vivo*. While their system was built for minimally invasive surgeries, imaging was performed during open surgery. Zhang et al.^65^ used a similar system to identify different types of tissues. They identified an improvement in classification accuracy using multispectral images over using RGB images. However, their system was tested on *ex vivo* tissues and not during live surgery. Some researchers built laparoscopic systems with dual channels, such that both live video and spectral images can be simultaneously captured. This was commonly done with the use of beam splitters. Köhler et al.^70^ developed a spatial laparoscopic camera for aiding esophagus surgery that captured monochromatic video and spectral images. To validate their system, they compared it with a commercial spectral camera developed for surgical settings. The specimen used was *ex vivo* human esophagus with adenocarcinoma. They found that their own compact laparoscopic system showed carcinoma classification result consistent with that of commercial devices. A similar dual camera endoscopic system that showed both spectral image and real-time video was developed by Yoon et al.^231^ They demonstrated the system using an *ex vivo* pig esophagus and used linear unmixing to estimate concentration of staining solution (see Fig. 15). They stained the esophagus with methylene blue (MB) solution and used measured absorbance to calculate the MB concentration throughout the tissue. Yoon et al.^276^ later improved the system and used it for clinical testing on 10 patients that underwent colonoscopy. They used spectral angle mapping to extract features and k-nearest neighbors to classify between normal mucosa and polyps.

#### 7.2.2 Spectral surgical microscope

A surgical microscope or operating microscope is a system of optical microscopy designed for aiding surgical procedures. They are useful to the point of necessity in microsurgery.^206^ The use of microscopy in surgical theaters has been around since the late 17th century. Today, surgical microscopes are used in many types of surgeries, ranging from micro-operations, such as dentistry, neurosurgery, and ENT surgery, to macro-operations, such as spine surgery, tumor resection, and plastic and reconstructive surgery. Modern surgical microscopes are engineering marvels, combining high-power optics, precise maneuverability, and good stability. They also have many digital components, which allow combination with many imaging modules, such as fluorescence imaging, optical coherence tomography, laser speckle imaging, and spectral imaging. Spectral imaging is of special interest to many researchers, because the noninvasive and noncontact nature of the technology means spectral imaging can provide visual aids with minimal complications. Furthermore, surgical microscopes often provide light sources sufficient for both the surgical operation and the image acquisition in the forms of xenon light, halogen light, and LED. This reduces the need for additional illumination as seen in other acquisition setups.

Many setups for spectral imaging in surgical microscope used a monochrome imaging camera, a broadband light source, and variable filters/filter wheel. Van Brakel et al.^61^ used an LCTF for the filters in their setup. They used the system to create high-resolution images of a dental implant; more specifically, they used the spectral data to estimate the soft tissue thickness and height surrounding the implant. To do this, they used a model of absorbance based on mucosa thickness. Postprocessing was required to align spectral images due to the motion blur. Nevertheless, the system was able to estimate soft tissue thickness consistent with previous literature. Both Roblyer et al.^277^ and Martin et al.^278^ used filter wheels for their acquisition setup. For illumination, Roblyer et al. replaced the original light sources of the surgical microscope with mercury lamp. While Martin et al. used a monochromatic camera, Roblyer et al. used an RGB camera and used the raw images to perform processing on. Pichette et al.^64^ used an ultracompact camera with a surgical microscope. The system used 4×4 SRDA to detect 16 spectral bands in the range 481 to 632 nm. With the system, they segmented blood vessels, assessed hemoglobin concentration, and detected potential vasomotion and epileptic spike responses.

## 8 Discussion and Future Directions

Spectral imaging, which includes both multispectral and hyperspectral imaging, acquires images in many wavelengths and beyond the visible light range. The vast amount of data acquired both spatially and spectrally offer benefits in understanding biochemical compositions of tissues and their locations. The main drive for designing compact and lightweight spectral cameras came from remote sensing, where small cameras are necessary to fit onto UAVs. However, many other imaging-intensive fields, such as biomedical imaging, benefited tremendously from this progress in the development of compact systems. We reviewed the technological progress made in the engineering and manufacturing of compact spectral cameras and found that current compact systems are vastly superior compared with many of their bulky counterparts of only 20 years ago. While the engineering principles have existed since the mid-20th century, it was manufacturing progress that drove the miniaturization of many previously cumbersome systems. For example, the physics behind FPI systems has been known since the 17th century. However, manufacturing them in large quantity and small size required lithography, which has only been possible since the 1990s. As such, we expect that future progress in generating more compact cameras will come from new manufacturing techniques. We also expect future researchers will focus on the engineering and design of compact snapshot cameras. Compared with spatial and spectral scanning cameras, snapshot cameras have a smaller number of mechanical components, which makes them a prime candidate for miniaturization. The variety of methods available for capturing snapshot images meant that researchers could pursue different paths toward miniaturization as well. As of writing this, some of the smallest existing spectral cameras (<30  g) were all snapshot cameras using SRDA technology.^53^^,^^54^^,^^76^^,^^161^^,^^279^

### 8.1 Hardware Limitations and Potential Solutions

Currently, there is still a large tradeoff in spatial scanning, spectral scanning, and snapshot imaging cameras. The tradeoff has three main components: spatial raster resolution, spectral number resolution, and acquisition time. The spatial and spectral scanning systems are optimized for spatial and spectral resolution, whereas the snapshot scanning systems are being optimized for acquisition time. If there are any “dream” spectral imaging systems that can achieve large spatial and spectral resolution within a short amount of time, several engineering barriers must be overcome. First, what should the acquisition mechanism be? If the system is snapshot, it needs to record the entire datacube onto the image sensor. The sensor for such a system would be much larger than any counterpart RGB imaging sensors; this already can impact the size of the camera. If the system is spatial or spectral, then the mechanism for translating or moving the filter must be fast enough to scan through the entire field in a reasonable time. Second, how will data recording work? Due to the enormous amount of data spectral cameras capture, most systems, even commercial ones, send raw data and receive commands to and from another computer. Behmann et al.^144^ described a commercial system that performed live calibrations without user input. New generations of spectral camera systems are heading toward on-the-spot image processing within the system hardware, which can make analysis much more seamless.

We briefly touched upon the power issues of existing cameras. For now, power consumption is not important because many laboratory-based systems draw power from the grid. However, in a future where biomedical spectral imaging systems will be used in low-resource situations, remote power sources and low power consumption must be a research priority. Another potential problem with compact spectral cameras is system cooling. Thermal radiation emitted by the instrument itself is negligible in the visible-NIR range but can affect the imagers working in the long wave infrared (LWIR, 8000 to 14,000 nm) range.^280^ To minimize this effect, LWIR spectral cameras need a specific cooling apparatus, which increases the size and weight of the system.

### 8.2 Quest for Lower Costs

A barrier toward popularization of spectral imaging research is the high cost of commercialized systems. As of now, a commercial spectral imaging camera can cost tens of thousands of dollars, many orders of magnitude more than their commercialized RGB counterparts. The reason for this is due to the small market size and complex manufacturing process. Manufacturing accurate spectral imaging systems requires precise spectral components and elaborate calibration processes, similar to the manufacturing process of other precision instruments. To counter this, many research facilities and hobbyists have produced customized spectral cameras using off-the-shelf custom instruments. We reviewed the use of open-source hardware and software, 3D printing, smartphone, and low-cost spectroscopy, and off-the-shelf optical systems. One common thread that enables all of them is the rise of personal simulation and modeling software. Ray-tracing software, optic simulation software, and 3D modeling software make customization possible. Still, a wide gap exists between the quality of COTS systems and that of commercialized systems. Many commercialized methods used high precision manufacturing standards. For example, SRDA, MEMS, and Offner spectrometers used lithographic manufacture. These methods were limited to research labs and clean room operations.^121^^,^^166^ If systems are built using low-cost components, extensive optical calibrations are needed, typically using a secondary spectral camera or a known light source. Improper calibration severely distorts the experiment outcomes, especially in applications that require high spectral and spatial precision.

Progress in making more low-cost customized spectral cameras will come from engineering using off-the-shelf components and from the open-source movement. Researchers should share new systems designs through open-access journals and invite collaboration and improvements. While open-source design is becoming increasingly accepted, it faces significant challenges in medical research. Many new designs did not undergo the strict regulation necessary for medical devices. They also had lackluster business models, which discouraged further developments. Copyright laws that cover open-source designs differ between hardware and software. Software is “created work” that is often legally protected under copyright, whereas hardware is “invention” that is protected under patent. Many companies that manufacture open-source hardware still protect their product under a trademark, which acts as a form of quality assurance.^281^ 3D printing and open-source movements have intertwined roots; software used to design and model in 3D are free and have large exchange forums on the internet. The majority of individuals involved in the 3D printing community are also involved in open-source projects, which shows a mindset of collaboration.^282^ We summarized the use of 3D printing for the manufacturing of customized parts and housing of compact spectral imagers. However, the biggest issue with 3D-printed housing is the mechanical durability. PLA, the most commonly used material for 3D printing, is cheap and accurate but has low heat tolerance and outdoor tolerance. It also has the potential to shrink after cooling. Future researchers interested in developing 3D-printed housing for hyperspectral cameras should study different materials and their durability; many materials exist such as acrylonitrile butadiene styrene, carbon fiber filaments, or metal-filled filaments that can serve as an alternative to PLA.

We believe that smartphones will be an important component of future compact low-cost spectral imagers. Smartphones are already engineered to be extremely compact with good processing and low power consumption. They are also relatively low-cost and widely available. Smartphone cameras have achieved considerable progress in the last decades. Researchers should use smartphones not just as a camera substitution but also as a control unit. For this, open-source phone operating systems should be preferred, because they allow control software to be written and shared freely. Smartphones are also interconnected to the communication network, which means that they can be used in a larger IoT system. We expected more smartphone-based systems to be used in low resource settings or in coordinated clinical trials. A potential problem is that smartphones have a variety of different configurations short-term technical support, so long-term development and collaboration can be difficult.

### 8.3 Future of Spectral Cameras in Bioimaging

In the second part of the paper, we reviewed the use of compact spectral imagers in the biomedical imaging field. Building spectral imaging biomedical systems in many cases were similar to “plug-and-play” systems. We believe that compact imaging systems will become much more common in clinical settings. The smaller size means that there will be less space, which means more room for other instruments. We predict that compact spectral cameras in biomedical imaging will have two types of applications. First, they will replace existing bulky spectral imaging systems. Surgery and diagnostic devices will be the main beneficiary of this change. In surgery, smaller cameras can be fitted on top of endoscopes, surgical microscopes, and operating robots without much interference to the surgical process. Already, spectral imaging has enabled the visualization of key features during surgery but it has not been widely adopted because of bulky sizes.^7^ Compact spectral cameras can allow for widespread adoption of spectral imaging technologies in the surgery room. We believe that snapshot cameras will be the dominant system in these situations because of their rapid acquisition time. Compact spectral systems will be used for more diagnosis of skin and retinal diseases *in vivo*. The strength of spectral devices comes from the fact that they are noninvasive and fast, which means that sensitive organs such as the retina and skin are prime candidates for diagnosis. These organs also contain well-known spectral-sensitive information, most notably blood oxygenation rate. The lighter weights will enable some systems to go from tabletop to handheld, which means that they can be much more convenient for clinical settings.

Second, compact spectral cameras will be used to research new physiological processes. If we want to see more research being done in biomedical imaging using compact cameras, we must understand more about the spectral signatures of physiological processes. Knowledge of hemoglobin spectra and skin physiology has already helped researchers to construct elaborate models to diagnose diseases such as melanoma, burns, wounds, ulcer, diabetic foot, and erythema. Large pathology datasets have been used to construct classification algorithms for digital staining, cancerous cell diagnosis, and cellular segmentations.^283^ We expected similar progress will be made in the retina, as it is currently linked to many complex diseases of the central nervous system.^284^ Recently, new understanding of how amyloid beta affects the scattering profile led to the development of hyperspectral imaging for Alzheimer’s diagnosis.^56^^,^^274^^,^^57^ We advocate for creations and sharing of hyperspectral imaging databases. Currently, most spectral imaging databases are satellite images used for remote sensing. Only a handful of hyperspectral databases are for biomedical purposes,^285^^,^^286^ and they are of specific diseases. Creation of new databases is difficult: acquisition requires a hyperspectral camera system and many patients or specimens. They also require a large hosting space, which can be hundreds of gigabytes. However, the scientific contribution of such database will be invaluable if they advance our understanding of human physiology.

We also predict the use of compact spectral imaging alongside other imaging modalities. Spectral imaging is not always the superior imaging modality. New applications are limited by the penetration depth of light through the skin tissues. Light in the VIS-NIR region has a penetration depth ranging from 0.48 mm at 550 nm to 3.57 mm at 850 nm. For comparison, photoacoustic multispectral imaging can achieve a penetration depth of up to 5 cm with a handheld system.^20^ To circumvent this shortcoming, imaging in the SWIR wavelengths has been proposed to provide greater penetration depths.^88^ By combining compact spectral imagers with other imaging modality, the multimodal system was constructed to provide point-of-care analysis to the patients.^108^^,^^225^ There are several different imaging modalities that pair well with hyperspectral imaging systems. Optical coherent tomography (OCT) captures images with depth of several millimeters and micrometer resolution. Combination of OCT and spectral imaging provided both surface chemical information and depth information.^287^^,^^288^ LSCI provided dynamic movements information of blood vessels, which paired well with spectral imaging ability to resolve blood oxygenation contents.^289^ Raman spectroscopy provided detailed chemical information in a small spatial scale, which complemented spectral imaging.^290^ Compact devices exist for many of these modalities, which means that multimodal systems can still be compact and convenient.

## 9 Conclusion

From large and bulky systems that were used on satellites and aircraft for remote sensing, spectral cameras now exist as compact and portable systems. Both the technology and the applications sides of spectral imaging remained not fully developed. New manufacturing methods and advances in computational speed mean that spectral cameras of the future can be high-quality, fast, and compact without sacrificing the cost factor. Spectral cameras becoming more compact and low-cost means that more individuals can use them to benefit biomedical research.
